# The Effect of Lifestyle Interventions on Anxiety, Depression and Stress: A Systematic Review and Meta-Analysis of Randomized Clinical Trials

**DOI:** 10.3390/healthcare12222263

**Published:** 2024-11-13

**Authors:** Sohrab Amiri, Nailah Mahmood, Syed Fahad Javaid, Moien AB Khan

**Affiliations:** 1 Spiritual Health Research Center, Lifestyle Institute, Baqiyatallah University of Medical Sciences, Tehran 17166, Iran; rsr.amiri.s@bmsu.ac.ir; 2 Division of Health Research, Lancaster University, Lancaster LA1 4YW, UK; n.mahmood2@lancaster.ac.uk; 3 Health and Wellness Research Group, Department of Psychiatry and Behavioral Sciences, College of Medicine and Health Sciences, United Arab Emirates University, Al-Ain 15551, United Arab Emirates; 4 Health and Wellness Research Group, Department of Family Medicine, College of Medicine and Health Sciences, United Arab Emirates University, Al-Ain 15551, United Arab Emirates

**Keywords:** lifestyle, anxiety, depression, stress, mental disorders, systematic review, meta-analysis

## Abstract

Background/Objectives: Depression, anxiety, and stress are common mental health issues that affect individuals worldwide. This systematic review and meta-analysis examined the effectiveness of various lifestyle interventions including physical activity, dietary changes, and sleep hygiene in reducing the symptoms of depression, anxiety, and stress. Using stress as an outcome and conducting detailed subgroup analyses, this study provides novel insights into the differential effects of lifestyle interventions across diverse populations. Methods: Five databases were systematically searched: PubMed, Web of Science, Scopus, Cochrane Library, and Google Scholar, for gray literature searches. Keywords were used to search each database. The search period was from the conception of the databases until August 2023 and was conducted in English. For each analysis, Hedges’ g was reported with a 95% confidence interval (CI) based on the random-effects method. Subgroups were analyzed and heterogeneity and publication bias were examined. Results: Ninety-six randomized clinical trial studies were included in this meta-analysis. Lifestyle interventions reduced depression (Hedges g −0.21, 95% confidence interval −0.26, −0.15; *p* < 0.001; *I*^2^ = 56.57), anxiety (Hedges g −0.24, 95% confidence interval −0.32, −0.15; *p* < 0.001; *I*^2^ = 59.25), and stress (−0.34, −0.11; *p* < 0.001; *I*^2^ = 61.40). Conclusions: Lifestyle interventions offer a more accessible and cost-effective alternative to traditional treatments and provide targeted benefits for different psychological symptoms.

## 1. Introduction

Mental disorders are among the conditions that place a high burden on healthcare [1] and remain among the primary causes of disease burden worldwide. However, there is no evidence of a decrease in this burden compared to previous decades [2]. Depression and anxiety are two categories of common mental disorders with a significant health burden [2]. In 2021, the Global Burden of Disease Study indicated that depression and anxiety are leading causes of disability, and both are among the 25 leading causes of disability worldwide [2,3]. The global prevalence of depressive disorders in 2019 was equal to 3440.1 per 100,000, and the prevalence rates for men and women were 2713.3 and 4158.4 per 100,000, respectively [2]. In addition, the prevalence of anxiety disorders is 3379.5/100,000, and the prevalence rates for men and women are 2859.8 and 4694.75 per 100,000, respectively [2]. The COVID-19 pandemic has affected public health as well as social structures, contributing to a significant increase in stress levels and further exacerbating mental health challenges worldwide [4,5].

According to a World Health Organization (WHO) report published in 2022, one out of every eight people worldwide live with a mental illness [6]. Thus, globally, 970 million people live with a mental disorder, of which depression and anxiety are the most common [7]. Several factors are associated with the prevalence of depression, anxiety disorders, and stress, including personality traits [8], financial status [9], biological factors [10], parental factors [11], and chronic diseases [12]. Additionally, a class of factors that can affect depression, anxiety, and stress is related to lifestyle, including physical activity [13,14,15,16,17], dietary patterns [18,19], sleep problems [20,21], smoking [22,23], and body mass index [24,25,26].

Lifestyle refers to “the characteristics of inhabitants of a region in special time and place” [27]. Interventions based on a healthy lifestyle have improved physical and mental health, with several studies exhibiting their effectiveness in cases of type 2 diabetes [28,29], obesity [30,31], cardiovascular risk [32], reducing cancer risk [33], obstructive sleep apnea [34], preventing weight gain [35], and mental health [36]. Considering the role of lifestyle interventions in improving health status, studies have investigated their effects in improving mental health and issues related to it [37,38].

A healthy lifestyle effectively reduces depression and anxiety [39,40]. A comprehensive review of studies conducted on the effectiveness of lifestyle interventions for common mental disorders, including depression and anxiety, indicated that there is extensive research available on this field, based on which, review and meta-analysis studies have also been conducted [39,40,41,42,43,44,45,46]. Specifically, for depression, results indicate that Cohen’s effect size ranges from −0.18 to −0.95 [40,41,43] while, for anxiety symptoms, Cohen’s effect size has been reported at −0.19 [39]. Although these reviews and meta-analyses offer valuable insights, they have revealed critical gaps. Most studies have focused on depression and anxiety; however, psychological stress, which is a distinct mental health condition, has not been thoroughly explored [47]. Although the effectiveness of lifestyle interventions may vary across different patient populations, few studies have analyzed the outcomes based on these differences. Given that depression and anxiety are more prevalent among women, the specific impact of lifestyle interventions on these disorders in women has been understudied [48]. Furthermore, various scales have been used to measure depression, anxiety, and stress; however, the potential impact of these differences in measurement tools on study outcomes has not been sufficiently addressed in previous meta-analyses. Recognizing these gaps, this systematic review and meta-analysis aimed to evaluate the effects of lifestyle interventions on depression, anxiety, and stress. Additional objectives include analyzing the influence of these interventions across different population subgroups, specifically among women, and investigating how measurement scales may affect the results.

## 2. Methods

### 2.1. Inclusion and Exclusion Criteria

1. The population studied in this research were under lifestyle interventions.
2. Eligible studies must have a lifestyle intervention group and a control group.
3. The outcomes examined in these studies were depression, anxiety, and stress.
4. Randomized clinical trials were eligible. Non-randomized clinical trials, quasi-experimental, and cluster randomized clinical trials were not eligible. Quasi-experimental studies were excluded because of the likelihood of confounding variables affecting the internal validity. Furthermore, studies presenting mixed or combined results were omitted to maintain uniformity in the outcome metrics for depression, anxiety, and stress. It was not possible to calculate the exact effect size, because the studies did not report the number of clusters, intra-class correlation was not reported in cluster randomized control trials, and it was not possible to estimate the effect size correctly [49,50], nor pre-post designs.
5. Studies that studied mixed multiple outcomes were not eligible.
6. For some studies. several reports were published and, in these cases, only the study with the best quality was included in the meta-analysis, excluding the rest.

### 2.2. Information Sources

Five databases were systematically searched to retrieve eligible articles: PubMed, Web of Science, Scopus, the Cochrane Library, and Google Scholar. Google Scholar was specifically used to identify gray literature. A set of keywords were used to search each database. In addition, all references for previous review studies in this regard were also searched by one of the authors. The search period was demarcated from the beginning of database formation. The search was conducted in English until August 2023.

### 2.3. Search Strategy

**Figure 1:**
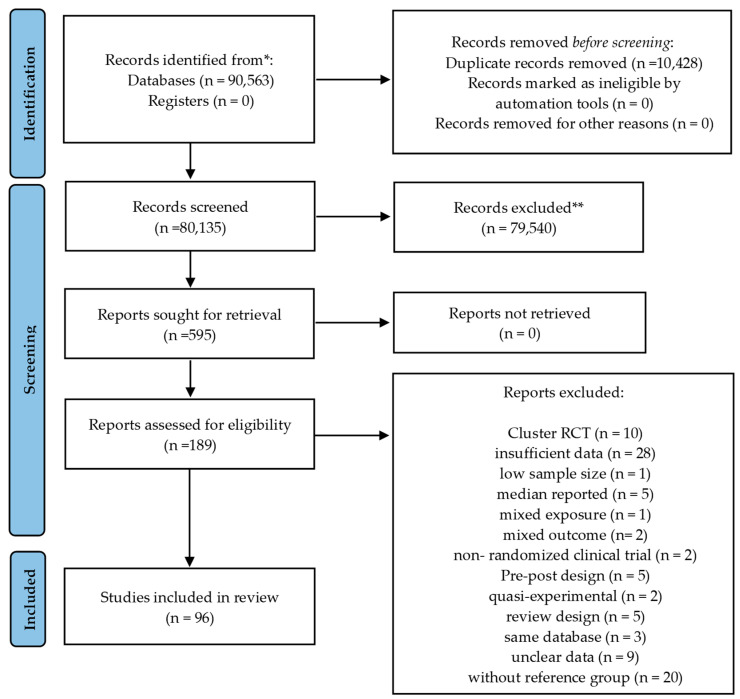
Flowchart diagram of screening studies included in this meta-analysis [51]. * Consider, if feasible to do so, reporting the number of records identified from each database or register searched (rather than the total number across all databases/registers). ** If automation tools were used, indicate how m7 any records were excluded by a human and how many were excluded by automation tools.

The review protocol has been registered with PROSPERO under the identifier CRD42023390131. Since the data used in this review were gathered from publicly accessible databases and online searches, ethics committee approval and informed consent were not required necessary. The study selection process is illustrated in Figure 1. A syntax of keywords is shown in Appendix A.

### 2.4. Selection Process

Eligible studies were screened and selected as follows. First, each author screened the collection of studies compiled from five databases based on the inclusion and exclusion criteria. Then, the work was divided into eligible articles, where each author reviewed a set of articles. This process was performed independently. Disagreements were resolved by discussing the final articles.

### 2.5. Data Collection Process

All eligible articles were divided among the authors so that each of them could extract the necessary data. After extracting data from each study, all extracted data were checked again. If the study data were insufficient, one of the authors contacted the other to obtain the necessary information.

### 2.6. Data Items

The intervention variable used in the present study consisted of interventions based on lifestyle. For an intervention to be considered as “lifestyle” oriented, at least two components from, the total lifestyle items had to be included. The outcomes of the study were depression, anxiety, and stress. Depression and anxiety are common mental health disorders. Instruments to measure depression, anxiety, and stress were used in this study. All scales used are listed in Table 1.

### 2.7. Study Risk of Bias Assessment

Following PRISMA guidelines, we used the Cochrane Collaboration’s risk of bias tool to evaluate the quality of the included studies [51]. The tool includes five dimensions of quality assessment: selection bias, performance bias, detection bias, attrition bias, and reporting bias. Bias was evaluated by judging each element from the five key domains. Each element was classified as having high, low, or unclear risk of bias. In this qualitative evaluation, the authors entered independently, and then qualitative evaluations were integrated through a discussion of disagreements. Overall, the quality was sufficient to support robust conclusions, with most studies meeting acceptable quality standards. A detailed summary of the risk of bias for each study is provided in Table 1, which aids in interpreting the reliability and generalizability of the meta-analysis findings.

### 2.8. Effect Measures

The effect size used in this study was the standardized mean difference, which was reported in the form of Hedges’ g effect size and 95% confidence interval (CI). Means, standard deviations, and sample sizes were used for each intervention and control group. In cases where these statistics were not reported, the sample size and *p* value were used.

### 2.9. Synthesis Methods

To calculate the effect size, the mean, standard deviation, and sample size of the intervention and treatment groups were extracted in the post-test. In some studies, instead of standard deviation, standard error or confidence interval was reported, and the Cochrane Handbook procedures were used to convert these into standard deviations [52]. Some studies used the mean change or mean difference or other tests to check for differences between the intervention and control groups. In this case, existing procedures were used to calculate the effect size, which included the use of sample size, *p* value, and direction, the details of which are mentioned in the guide [53]. Some studies have reported multiple dependent outcomes, which were transformed using existing procedures using Comprehensive meta-analysis-Version 3.3 software [53,54]. The effect size used in this study was Hedges’ g, and the 95% confidence interval was classified as follows: 0.20 (low), −0.50 (medium), 0.80 (large) [55]. For each analysis, Hedges’ g was reported with a 95% confidence interval (CI) based on the random-effects method. Hedges’ g was used because it considers the sample size, and the studies included in this meta-analysis had different sample sizes [56]. Based on the goals of this study, the following analyses were conducted. The effects of lifestyle interventions on depression, anxiety, and stress were analyzed separately. For each of these analyses, several subgroups were created based on the type of population and scale used to measure depression, anxiety, stress, and sex. Heterogeneity in the studies included in the meta-analysis and publication bias were also examined. These tests were used for the heterogeneity Q test and *I*^2^ [57,58]. An interpretation of *I*^2^ is as follows: may not be important, moderate, substantial, and considerable [59]. For publication bias, these tests used funnel plots [60,61], Egger’s test [62,63], and Trim and fill [64]. Comprehensive meta-analysis-3 software was used in this study [53].

## 3. Results and Discussion

### 3.1. Screened Studies

The studies were screened based on the flowchart shown in Figure 1. After identifying duplicate studies, ineligible studies and other studies that did not meet the inclusion criteria were excluded. Finally, 97 clinical trial studies [65,66,67,68,69,70,71,72,73,74,75,76,77,78,79,80,81,82,83,84,85,86,87,88,89,90,91,92,93,94,95,96,97,98,99,100,101,102,103,104,105,106,107,108,109,110,111,112,113,114,115,116,117,118,119,120,121,122,123,124,125,126,127,128,129,130,131,132,133,134,135,136,137,138,139,140,141,142,143,144,145,146,147,148,149,150,151,152,153,154,155,156,157,158,159,160] were included in this meta-analysis.

**Table 1:** Studies included in meta-analysis.

Author and Year	Country	Follow-Up	Population	Age	Sex % Women	Sample Size	Lifestyle Intervention Definition	Mental Disorders	Mental Disorders Scoring	Measure	Quality Dimensions						Results N (Mean, Standard Deviation)
											Selection Bias		Performance Bias	Detection Bias	Attrition Bias	Reporting Bias	
											Random Sequence Generation	Allocation Concealment					
Anderson 2015 [66]	Australia	3-month	Breast cancer	45–60	Women	51	Pink Women’s Wellness Program	1-Depression 2-Anxiety	Higher scorer indicating more mental problemss	1-Greene Climacteric Scale	Low	Unclear	High	High	Low	Unclear	Depression Intervention 26 (4.1 ± 2.1) Control 25 (4.3 ± 3.6) Anxiety Intervention 26 (4.9 ± 3.5) Control 25 (4.5 ± 2.8)
Azami 2018 [67]	Iran	3-month 6-month	Type 2 Diabetes	≥18	65.5% women	142	Nurse-led diabetes self-management	1-Depression	Higher scorer indicating more mental problemss	1-Center for Epidemiologic Studies Depression Scale	Low	Low	High	Low	Low	Unclear	3-month Intervention 71 (11.98 ± 4.97) Control 71 (12.84 ± 4.61) 6-month Intervention 71 (11.95 ± 5.03) Control 71 (12.91 ± 4.5)
Brennan 2012 [68]	Australia	6-month	Overweight/Obese	11–19	54% women	63	Cognitive Behavioural Lifestyle	1-Depression 2-Anxiety 3-Stress	Higher scorer indicating more mental problemss	1-Depression anxiety and stress scale	Low	Unclear	Unclear	Low	Low	Unclear	Depression Intervention 40 (7.9 ± 9.6) Control 21 (4.2 ± 5.9) Anxiety Intervention 40 (7.0 ± 7.4) Control 21 (6.8 ± 6.0) Stress Intervention 40 (9.9 ± 10.2) Control 21 (7.7 ± 7.3)
Bringmann 2022 [69]	Germany	1-month 2-month 6-month	Mild to moderate depression	49.1 ± 11.1 in intervention 51.0 ± 12.7 in control	77.8% women	54	Meditation-Based Lifestyle Modification	1-Depression 2-Stress	Higher scorer indicating more mental problemss	1-Beck Depression Inventory 2-Perceived Stress Scale-10	Low	Low	Low	Low	Low	Unclear	Depression 1-month Intervention 27 (16.81 ± 10.65) Control 27 (20.89 ± 8.14) 2-month Intervention 27 (13.59 ± 10.63) Control 27 (21.59 ± 9.67) 6-month Depression Intervention 27 (13.68 ± 10.36) Control 27 (20.80 ± 10.95) Stress 2-month Intervention 27 (20.11 ± 5.34) Control 27 (27.15 ± 4.58) 6-month Intervention 27 (20.54 ± 5.64) Control 27 (25.85 ± 6.59)
Brown 2001 [70]	USA	8-week	Mild to moderate depression	19–78	Women	104	Multi-Modal Intervention	1-Depression	Higher scorer indicating more mental problems	1-Center for Epidemiologic Studies Depression Scale	High	Low	Unclear	Unclear	Low	Unclear	Depression Intervention 53 (10.4 ± 7.3) Control 51 (16.7 ± 10.4)
Casañas 2012 [72]	Spain	3-month 6-month 9-month	Major depression	≥20	89.2% women	231	Psycho-educational	1-Depression	Higher scorer indicating more mental problems	1-Beck Depression Inventory	Low	Low	High	High	Low	Unclear	3-month Intervention 119 (15.42 ± 7.53) Control 112 (17.54 ± 7.18) 6-month 119 (15.37 ± 8.74) Control 112 (16.51 ± 7.60) 9-month 119 (15.09 ± 8.62) Control 112 (16.35 ± 7.84)
Cezaretto 2012 [73]	Brazil	9-month	Type 2 diabetes	18–79	67.8% women	177	Intensive interdisciplinary intervention	1-Depression	Higher scorer indicating more mental problems	1-Beck Depression Inventory	Unclear	Unclear	Unclear	Unclear	High	Unclear	Intervention 75 (8.4 ± 7.7) Control 60 (5.2 ± 5.1)
Chang 2018 [74]	Korea	3-month	Older Adults with major depressive disorder	77.8 ± 6.6	87.1% women	93	Multi-Domain Lifestyle Modification	1-Depression	Higher scorer indicating more mental problems	1-Geriatric Depression Scale (GDS)-Short Form	Low	Low	Unclear	Low	Low	Unclear	Intervention 47 (7.5 ± 4.1) Control 46 (10.2 ± 3.6)
Charandabi 2017 [75]	Iran	2-month	spouses of pregnant women	31.9 ± 5.3	Men	126	Life Style Based Education	1-Depression 2-Anxiety	Higher scorer indicating more mental problems	1-Edinburgh Postnatal Depression Scale 2-Spielberger’s State-Trait Anxiety Inventory	Low	Low	High	Low	Low	Unclear	Depression Intervention 62 (2.7 ± 3.4) Control 63 (4.3 ± 3.8) State anxiety Intervention 62 (30.1 ± 7.7) Control 63 (35.8 ± 10.5) Trait anxiety Intervention 62 (30.7 ± 7.6) Control 63 (35.8 ± 9.7)
Chiang 2019 [76]	Taiwan	3-month	Metabolic Syndrome	≥40	Women	68	Lifestyle modification combined with motivational counseling	1-Depression	Higher scorer indicating more mental problems	1-Beck Depression Inventory	Low	Low	Unclear	Low	Unclear	Unclear	Intervention 34 (3.8 ± 1.5) Control 34 (9.1 ± 6.9)
Clark 2012 [77]	USA	6-month	Older people	60–95	65.9% women	360	Lifestyle intervention (Well Elderly Lifestyle)	1-Depression	Higher scorer indicating more mental problems	1-Center for Epidemiologic Studies Depression Scale	Low	Unclear	Low	Low	Low	Unclear	Intervention 186 (12.47 ± 9.68) Control 173 (13.53 ± 11.17)
Croker 2012 [78]	UK	6-month	Obese	10.3 ± 1.6	69.4% women	63	family-based behavioral treatment	1-Depression	Higher scorer indicating more mental problems	1-Children’s Depression Inventory	Low	High	High	Low	Unclear	Unclear	Intervention 33 (49.24 ± 6.91) Control 30 (48.13 ± 6.97)
Desplan 2014 [79]	France	1-month	obstructive sleep apnea	35–70	Unknown	22	lifestyle intervention	1-Depression 2-Anxiety	Higher scorer indicating more mental problems	1-Hospital anxiety and depression scale	Unclear	High	Unclear	Unclean	Unclean	Unclean	Depression Intervention 11 (4.7 ± 2.6) Control 11 (8.3 ± 3.6) Anxiety Intervention 11 (7.1 ± 3.7) Control 11 (10.4 ± 3.8)
Devi 2014 [80]	UK	6-week	Angina Population	66.27 (8.35) in intervention 66.20 (10.06) in control	25.5% women	94	Activate Your Heart	1-Depression 2-Anxiety	Higher scorer indicating more mental problems	1-Hospital anxiety and depression scale	Low	Low	High	High	Low	Unclean	Depression Intervention 37 (2.00 ± 2.00) Control 42 (2.00 ± 4.25) Anxiety Intervention 36 (4.14 ± 3.50) Control 39 (4.87 ± 3.73)
Dodd 2016 [81]	Australia	28-week 36-week 4-month	overweight or obese	29.4 (5.4) for intervention 29.6 (5.4) for control	Women	2142	lifestyle intervention	1-Depression 2-Anxiety	Higher scorer indicating more mental problems	1-Edinburgh Postnatal Depression Scale-10 2-Spielberger’s State-Trait Anxiety Inventory	Low	Low	Unclear	Unclear	Unclear	Unclear	Depression 28-week Intervention 976 (6.28 ± 4.53) Control 957 (6.12 ± 4.75) 36-week Intervention 976 (5.83 ± 4.58) Control 957 (5.63 ± 4.72) 4-month Intervention 976 (5.34 ± 4.51) Control 957 (5.02 ± 4.30) Anxiety 28-week Intervention 976 (10.56 ± 3.56) Control 957 (10.48 ± 3.66) 36-week Intervention 976 (10.64 ± 3.62) Control 957 (10.41 ± 3.56) 4-month Intervention 976 (10.18 ± 3.64) Control 957 (10.14 ± 3.50)
Forsyth 2015 [82]	Australia	3-month	patients with depression and anxiety	18–84	Both (%Women is unknown)	63	lifestyle intervention	1-Depression 2-Anxiety 3-Stress	Higher scorer indicating more mental problems	1-Depression anxiety and stress scale	High	High	Unclear	Unclear	High	Unclear	Depression Intervention 32 (6.0 ± 6.2) Control 31 (5.9 ± 3.5) Anxiety Intervention 32 (3.5 ± 3.3) Control 31 (3.7 ± 3.5) Stress Intervention 32 (6.7 ± 5.1) Control 31 (8.0 ± 4.8)
Furuya 2015 [83]	Brazil	6-month	Patients following percutaneous coronary intervention	≥18	43.3% women	60	educational programme	1-Depression 2-Anxiety	Higher scorer indicating more mental problems	1-Hospital anxiety and depression scale	Unclear	Low	Low	Low	Unclear	Unclear	Depression Intervention 30 (5.1 ± 4.4) Control 30 (7.6 ± 4.1) Anxiety Intervention 30 (5.4 ± 4.8) Control 30 (4.7 ± 3.5)
Garcia 2023 [84]	Spain	2-month 6-month 12-month	treatment-resistant depression	≥18	69.2% women	65	lifestyle modification program	1-Depression	Higher scorer indicating more mental problems	1-Beck Depression Inventory-II	Low	Low	Unclear	Unclear	Low	Unclear	2-month Intervention 34 (17.34 ± 10.8) Control 31 (24.87 ± 14.2) 6-month Intervention 34 (16.85 ± 13.3) Control 31 (23.17 ± 17.3) 12-month Intervention 34 (19.87 ± 15.9) Control 31 (23.33 ± 15.3)
Giallo 2014 [85]	Australia	2-week 6-week	Postpartum	>18	Women	98	psychoeducational intervention	1-Depression 2-Anxiety 3-Stress	Higher scorer indicating more mental problems	1-Depression anxiety and stress scale	Low	Unclear	Low	Unclear	Low	Unclear	2-week Depression Intervention 39 (3.07 ± 4.05) Control 59 (5.24 ± 7.01) Anxiety Intervention 39 (1.80 ± 3.03) Control 59 (2.86 ± 3.96) Stress Intervention 39 (10.00 ± 6.18) Control 59 (11.87 ± 9.33) 6-week Depression Intervention 39 (3.85 ± 4.09) Control 59 (4.64 ± 5.23) Anxiety Intervention 39 (1.95 ± 3.20) Control 59 (2.34 ± 3.49) Stress Intervention 39 (9.95 ± 7.41) Control 59 (10.88 ± 9.12)
Glasgow 2006 [86]	USA	2-month	Type 2 diabetes	61.5 ± 11.3	50% women	301	lifestyle intervention	1-Depression	Higher scorer indicating more mental problems	1-Patient Health Questionnaire	Unclear	Unclear	Unclear	Unclear	Low	Unclear	Intervention 147 (5.5 ± 5) Control 152 (5.5 ± 5.3)
Guo 2021 [87]	China	3-month 6-month	Gestational Diabetes Mellitus	≥18	Women	320	Intensive Lifestyle Modification	1-Stress	Higher scorer indicating more mental problems	1-perceived stress scale	Low	Low	High	Low	Low	Unclear	3-month Intervention 160 (24.22 ± 7.93) Control 160 (24.53 ± 6.72) 6-month Intervention 160 (24.18 ± 7.33) Control 160 (24.60 ± 5.47)
Han 2020 [88]	Hong Kong	15-week	major depressive disorder	47.06 (9.54) in intervention 45.44 (8.25) in control	Both (%Women is unknown)	33	Dejian mind-body intervention	1-Depression	Higher scorer indicating more mental problems	1-Hamilton Psychiatric Rating Scale for Depression (HRSD) 2-Beck Depression Inventory	Low	Low	Low	Low	Unclear	Unclear	HRSD Intervention 17 (6.50 ± 4.31) Control 16 (9.75 ± 4.16)) BDI Intervention 17 (17.94 ± 12.70) Control 16 (24.79 ± 14.91))
Heutink 2012 [89]	The Netherlands	Post-intervention 3-month	spinal cord injury	≥18	30.06% women	61	Multidisciplinary cognitive behavioral program	1-Anxiety	Higher scorer indicating more mental problems	1-Hospital anxiety and depression scale	Unclear	Unclear	Unclear	Unclear	Low	Unclear	Post-intervention Intervention 31 (5.6 ± 3.6) Control 30 (5.7 ± 3.4) 3-month Intervention 31 (5.9 ± 3.6) Control 30 (5.6 ± 3.6)
Hilmarsdóttir 2021 [90]	Iceland	6-month	type 2 diabetes mellitus	25–70	63.3% women	30	Sidekick Health smartphone app	1-Depression 2-Anxiety	Higher scorer indicating more mental problems	1-Hospital anxiety and depression scale	Unclear	Low	High	Low	Unclear	Unclear	Depression Intervention 15 (3.3 ± 3.0) Control 15 (4.2 ± 4.6) Anxiety Intervention 15 (4.1 ± 3.8) Control 15 (5.5 ± 4.7)
Holt 2019 [91]	UK	3-month 12-month	Schizophrenia	≥18	49% women	412	structured education lifestyle program	1-Depression	Higher scorer indicating more mental problems	1-Patient Health Questionnaire	Low	High	High	Low	Unclear	Unclear	3-month Intervention 178 (10.3 ± 6.3) Control 180 (10.1 ± 7.1) 12-month Intervention 167 (9.9 ± 7.0) Control 173 (9.6 ± 6.6)
Hwang 2019 [92]	Korea	4-week	nurses employed	Unspecified	94.6% women	56	Stress-Management Program	1-Depression 2-Anxiety 3-Stress	Higher scorer indicating more mental problems	1-Patient Health Questionnaire 2-Generalized Anxiety Disorder-7 3-Perceived Stress Scale-10	Unclear	Unclear	Unclear	Unclear	Unclear	Unclear	Depression * Intervention 26 (6.46 ± 4.99) Control 30 (6.93 ± 4.98) Anxiety Intervention 26 (4.23 ± 4.38) Control 30 (5.40 ± 4.38) Stress Intervention 26 (18.50 ± 3.56) Control 30 (19.16 ± 3.56)
Ihle-Hansen 2014 [93]	Norway	12-month	Stroke	72.6 (11.2 in intervention 70.6 (13.6) in control	46.7% women	195	multifactorial risk factor intervention program	1-Depression 2-Anxiety	Higher scorer indicating more mental problems	1-Hospital anxiety and depression scale	Unclear	Unclear	Low	Low	Low	Unclear	Depression Intervention 98 (2.91 ± 2.63) Control 97 (3.49 ± 3.02) Anxiety Intervention 97 (3.10 ± 2.83) Control 97 (3.95 ± 3.50)
Imayama 2011 [94]	USA	12-month	overweight/obese postmenopausal women	50–75	Women	204	diet and/or exercise interventions	1-Depression 2-Anxiety 3-Stress	Higher scorer indicating more mental problems	1-Brief Symptom Inventory-18 2-Perceived Stress Scale	Low	Unclear	Unclear	Low	Low	Unclear	Depression Intervention 117 (46.2 ± 8.2) Control 87 (48.4 ± 9.6) Anxiety Intervention 117 (43.5 ± 6.4) Control 87 (45.3 ± 8.7) Stress Intervention 117 (2.66 ± 2.27) Control 87 (3.89 ± 2.75)
Inouye 2014 [95]	USA	6-month	at risk for diabetes	≥30	Both %Women is unknown	40	Lifestyle Intervention	1-Depression	Higher scorer indicating more mental problems	1- Center for Epidemiologic Studies Depression Scale	Unclear	Unclear	Unclear	Unclear	Unclear	Unclear	Intervention 22 (12.21 ± 10.97) Control 18 (13.52 ± 11.28)
Ip 2021 [96]	China	6-week 12-week	moderate to severe depression	≥18	83.9% women	31	group-based lifestyle medicine	1-Depression 2-Anxiety 3-Stress	Higher scorer indicating more mental problems	1-Patient Health Questionnaire 2-Depression anxiety and stress scale	Low	Low	High	Low	Low	Unclear	6-week PHQ-9-depression Intervention 16 (7.4 ± 2.3) Control 15 (9.5 ± 3.7) DASS—Depression Intervention 16 (7.0 ± 3.7) Control 15 (12.9 ± 7.8) Anxiety Intervention 16 (4.3 ± 2.5) Control 15 (11.1 ± 8.0) Stress Intervention 16 (13.5 ± 7.7) Control 15 (17.1 ± 9.6) 12-week PHQ-9-depression Intervention 16 (7.5 ± 3.6) Control 15 (10.2 ± 3.8) DASS—Depression Intervention 16 (9.5 ± 7.8) Control 15 (13.3 ± 9.0) Anxiety Intervention 16 (6.5 ± 2.8) Control 15 (10.7 ± 7.3) Stress Intervention 16 (11.5 ± 6.9) Control 15 (16.9 ± 9.6)
Jonsdottir 2015 [99]	Iceland	6-month	obstructive pulmonary disease	45–65	54% women	100	self-management programme	1-Depression 2-Anxiety	Higher scorer indicating more mental problems	1-Hospital anxiety and depression scale	Low	Unclear	Low	Unclear	Low	Unclear	Depression Intervention 46 (3.28 ± 3.30) Control 49 (3.92 ± 3.28) Anxiety Intervention 48 (6.60 ± 4.26) Control 52 (7.25 ± 3.61)
Kelly 2020 [100]	Australia	12-week 16-week	consumers of a community mental health service	18–65	58% women	43	peer delivered healthy lifestyle intervention	1-Depression	Higher scorer indicating more mental problems	1-Patient Health Questionnaire	Low	Low	Unclear	Low	Unclear	Unclear	12-week Intervention 13 (11.62 ± 6.55) Control 14 (12.79 ± 7.54) 16-week Intervention 16 (10.50 ± 6.13) Control 16 (11.94 ± 6.12)
Kieffer 2013 [101]	USA	Unknown	Pregnant	≥18	Women	275	Healthy Lifestyle Intervention	1-Depression	Higher scorer indicating more mental problems	1-Center for Epidemiologic Studies Depression Scale	Low	Low	Unclear	Low	Low	Unclear	Intervention 138 (11.24 ± 7.98) Control 137 (12.71 ± 7.84)
Kim 2011 [102]	Korea	12-week	Breast Cancer	26–69	Women	45	Matched Exercise and Diet	1-Depression 2-Anxiety	Higher scorer indicating more mental problems	1-Hospital anxiety and depression scale	Unclear	Unclear	Unclear	Unclear	Low	Unclear	Depression Intervention 23 (3.32 ± 2.58) Control 22 (5.85 ± 3.65) Anxiety Intervention 23 (3.97 ± 2.30) Control 22 (5.46 ± 2.76)
Koch 2021 [103]	Germany	12-week 24-week 48-week	Ulcerative Colitis	18–74	Unknown	97	Lifestyle Modification	1-Depression 2-Anxiety 2-Stress	Higher scorer indicating more mental problems	1-Hospital anxiety and depression scale 1-perceived stress scale	Low	Unclear	Unclear	Unclear	Low	Unclear	Depression 12-week Intervention 47 (4.74 ± 3.39) Control 50 (5.81 ± 3.91) 24-week Intervention 47 (5.57 ± 3.52) Control 50 (6.18 ± 3.59) 48-week Intervention 47 (4.45 ± 3.46) Control 50 (4.74 ± 3.36) Anxiety 12-week Intervention 47 (6.67 ± 3.84) Control 50 (7.45 ± 3.55) 24-week Intervention 47 (7.61 ± 4.26) Control 50 (7.55 ± 3.48) 48-week Intervention 47 (6.46 ± 3.98) Control 50 (6.55 ± 3.20) Stress 12-week Intervention 47 (14.00 ± 6.38) Control 50 (18.59 ± 6.89) 24-week Intervention 47 (15.76 ± 6.44) Control 50 (18.47 ± 6.29) 48-week Intervention 47 (13.75 ± 7.20) Control 50 (16.05 ± 6.80)
Kwon 2015 [105]	Korea	4-week	Community Dwelling	≥65	59.5% women	93	Wheel of Wellness counseling intervention	1-Depression	Higher scorer indicating more mental problems	1-Patient Health Questionnaire	Low	Low	High	Unclear	Unclear	Unclear	Intervention 43 (4.51 ± 4.59) Control 46 (5.02 ± 5.47)
Lee 2015 [106]	Korea	6-month	obstructive pulmonary disease	40–80	8.6% women	151	nurse-led problem-solving therapy	1-Depression	Higher scorer indicating more mental problems	1- Center for Epidemiologic Studies Depression Scale	Unclear	Unclear	Unclear	Unclear	High	Unclear	Intervention 78 (15.9 ± 8.0) Control 73 (17.2 ± 8.0)
Leemrijse 2016 [107]	the Netherlands	6-month	patients with recent coronary event	18–80	19% women	374	Hartcoach	1-Depression 2-Anxiety	Higher scorer indicating more mental problems	1-Hospital anxiety and depression scale	Low	Unclear	Low	Unclear	Low	Unclear	Depression Intervention 145 (3.31 ± 3.71) Control 167 (3.83 ± 3.64) Anxiety Intervention 145 (3.95 ± 3.59) Control 167 (4.88 ± 4.00)
Lund 2012 [108]	Norway	9-month	stroke survivors	75 (7.2 in intervention 79 (6.5) in control	51.2% women	204	lifestyle course	1-Depression 2-Anxiety	Higher scorer indicating more mental problems	1-Hospital anxiety and depression scale	Low	Low	Unclear	Low	Unclear	Unclear	Depression Intervention 39 (3.4 ± 2.7) Control 47 (4.2 ± 3.4) Anxiety Intervention 39 (3.1 ± 3.4) Control 47 (4.4 ± 4.0)
María Nápoles 2020 [109]	USA	3-month 6-month	breast cancer survivors	28–88	Women	153	stress management intervention	1-Depression 2-Anxiety 3-Stress	Higher scorer indicating more mental problems	1-Patient Health Questionnaire 2-Brief Symptom Inventory scales 3-Perceived Stress Scale	Low	Low	Unclear	Low	Low	Unclear	Depression 3-month Intervention 76 (6.81 ± 5.31) Control 77 (6.97 ± 5.12) 6-month Intervention 76 (6.96 ± 5.62) Control 77 (6.44 ± 5.15) Anxiety 3-month Intervention 76 (0.63 ± 0.61) Control 77 (0.65 ± 0.70) 6-month Intervention 76 (0.52 ± 0.53) Control 77 (0.70 ± 0.64) Stress 3-month Intervention 76 (14.45 ± 6.63) Control 77 (14.08 ± 7.35) 6-month Intervention 76 (14.70 ± 6.14) Control 77 (15.14 ± 6.28)
Martín 2014 [110]	Spain	6-month	Fibromyalgia	50.12 ± 9.07	93.5% women	110	Interdisciplinary PSYMEPHY Treatment	1-Anxiety	Higher scorer indicating more mental problems	1-Fibromyalgia Impact Questionnaire	Low	Unclear	Unclear	Unclear	Unclear	Unclear	Intervention 54 (13.41 ± 4.31) Control 56 (12.75 ± 4.55)
Mayer-Davis 2018 [111]	USA	18-month	Type 1 diabetes	13–16	38.8% women	99	Flexible Lifestyles for Youth	1-Depression	Higher scorer indicating more mental problems	1- Center for Epidemiologic Studies Depression Scale	Low	Low	Unclear	High	Low	Unclear	Intervention 118 (6∙63 ± 7∙12) Control 123 (8∙46 ± 7∙08)
Mensorio 2019 [112]	Spain	3-month	Obesity and hypertension	18–65	Unknown	106	Living Better	1-Depression 2-Anxiety 3-Stress	Higher scorer indicating more mental problems	1-Depression anxiety and stress scale	Low	Unclean	Unclear	Unclear	Low	Unclear	Depression Intervention 43 (2.88 ± 3.6) Control 48 (2.79 ± 3.5) Anxiety Intervention 43 (1.73 ± 2.6) Control 48 (3.04 ± 3.4) Stress Intervention 43 (3.40 ± 2.9) Control 48 (5.20 ± 4.1)
Michalsen 2005 [113]	Germany	12-month	Coronary Artery Disease	59.4 ± 8 8.6	22.8% Women	101	lifestyle modification program	1-Depression 2-Anxiety 3-Stress	Higher scorer indicating more mental problems	1-Beck Depression Inventory 2-Spielberger State-Trait Anger Expression Inventory 3-Cohen Perceived Stress Scale	Low	Low	Unclear	Unclear	Low	Unclear	Depression Intervention 48 (6.4 ± 4.2) Control 53 (7.6 ± 4.7) State anxiety Intervention 48 (36.5 ± 8.8) Control 53 (36.2 ± 7.6) Trait anxiety Intervention 48 (35.7 ± 8.3) Control 53 (37.5 ± 10.0) Stress Intervention 48 (19.1 ± 7.6) Control 53 (21.7 ± 7.7)
Moncrieft 2016 [144]	USA	6-month 12-month	Type 2 Diabetes	18–70	71.2% women	111	Lifestyle Modification	1-Depression	Higher scorer indicating more mental problems	1-Beck Depression Inventory-II	Low	Low	Unclean	Low	Low	Unclear	6-month Intervention 42 (10.75 ± 7.76) Control 48 (16.09 ± 9.15) 12-month Intervention 41 (9.85 ± 8.86) Control 46 (16.00 ± 10.80)
Moore 2011 [158]	Australia	6-month	At risk of type 2 diabetes	61.3 ± 11.1	59% women	307	group-based lifestyle intervention	1-Depression 2-Anxiety	Higher scorer indicating more mental problems	1-Depression anxiety and stress scale	High	Unclean	Unclean	Unclean	Unclean	Unclean	Depression Intervention 167 (5.32 ± 7.03) Control 85 (4.87 ± 6.78) Anxiety Intervention 167 (4.57 ± 5.84) Control 85 (3.56 ± 4.31)
Morales-Fernández 2021 [159]	Spain	3-month 6-month 9-month	non-malignant pain	45–61 percentile	67.7% women	279	nurse-led intervention	1-Depression 2-Anxiety	Higher scorer indicating more mental problems	1-Patient Health Questionnaire 2-Generalized Anxiety Disorder Scale	Low	Low	High	Low	Low	Unclear	Depression 3-month Intervention 174 (10.06 ± 5) Control 105 (11.31 ± 6.19) 6-month Intervention 174 (10.16 ± 5.11) Control 105 (11.61 ± 5.81) 9-month Intervention 174 (10.43 ± 5.29) Control 105 (12.66 ± 5.99) Anxiety 3-month Intervention 174 (8.43 ± 4.76) Control 105 (9.1 ± 5.31) 6-month Intervention 174 (7.72 ± 4.73) Control 105 (8.95 ± 4.92) 9-month Intervention 174 (7.51 ± 4.77) Control 105 (9.16 ± 4.7)
Moseley 2009 [115]	Australia	Post-Program 6-week	Adolescent	15.6 ± 0.6	66.7% women	81	School-Based Intervention	1-Depression	Higher scorer indicating more mental problems	1-Depression anxiety and stress scale	Unclear	Unclear	Unclear	Unclear	Unclear	Unclear	Post-Program Intervention 21 (10.0 ± 7.7) Control 12 (14.2 ± 11.8)) 6-week Intervention 17 (12.9 ± 7.3) Control 13 (13.9 ± 10.7)
Mountain 2017 [116]	UK	6-month 24-month	older adults	≥65	68.05% women	262	occupation-based lifestyle intervention	1-Depression	Higher scorer indicating more mental problems	1-Patient Health Questionnaire	Low	Low	High	Low	Low	Unclear	6-month Intervention 133 (3.8 ± 4.2) Control 122 (3.4 ± 4.3) 24-month Intervention 122 (3.8 ± 4.6) Control 114 (4.0 ± 4.8)
Murawski 2019 [117]	Australia	3-month 6-month	Adults with insufficient physical activity/poor sleep quality	18−55	80% women	160	physical activity and sleep quality	1-Depression 2-Anxiety 3-Stress	Higher scorer indicating more mental problems	1-Depression anxiety and stress scale	Low	Low	Unclear	Unclear	Low	Unclear	Depression 3-month Intervention 59 (10.6 ± 7.62) Control 65 (12.6 ± 7.97) 6-month Intervention 34 (10.9 ± 8.01) Control 53 (13.3 ± 9.49) Anxiety 3-month Intervention 59 (6.4 ± 3.65) Control 65 (7.5 ± 5.04) 6-month Intervention 34 (5.9 ± 3.54) Control 53 (8.9 ± 4.70) Stress 3-month Intervention 59 (13.6 ± 4.20) Control 65 (15.4 ± 4.97) 6-month Intervention 34 (13.0 ± 5.75) Control 53 (16.3 ± 5.24)
Nie 2019 [118]	China	3-month 6-month 9-month 12-month	coronary artery disease	18–80	27.5% women	284	lifestyle improving program	1-Depression 2-Anxiety	Higher scorer indicating more mental problems	1-Hospital anxiety and depression scale	Low	Low	High	Low	Low	Unclear	Depression 3-month Intervention 142 (9.85 ± 2.48) Control 142 (9.71 ± 2.94) 6-month Intervention 142 (9.44 ± 2.84) Control 142 (9.48 ± 3.06) 9-month Intervention 142 (8.57 ± 2.81) Control 142 (9.13 ± 3.22) 12-month Intervention 142 (8.21 ± 3.03) Control 142 (9.08 ± 3.30) Anxiety 3-month Intervention 142 (9.37 ± 2.74) Control 142 (9.63 ± 2.38) 6-month Intervention 142 (8.82 ± 2.51) Control 142 (9.21 ± 2.52) 9-month Intervention 142 (8.26 ± 2.24) Control 142 (8.93 ± 2.36) 12-month Intervention 142 (7.80 ± 2.38) Control 142 (8.88 ± 2.37)
O’Neill 2015 [119]	UK	6-month	prostate cancer	69.7 ± 6.8 in intervention 69.9 ± 7.0 in control	Men	94	diet and physical activity	1-Stress	Higher scorer indicating more mental problems	1-Perceived Stress Scale	Low	Low	Unclean	High	Low	Unclear	Intervention 47 (10.5 ± 6.9) Control 47 (11.2 ± 10.2)
O’Reilly 2016 [120]	Australia	12-month	Gestational Diabetes	≥18	Women	573	group-based lifestyle modification	1-Depression	Higher scorer indicating more mental problems	1-Patient Health Questionnaire	Low	Unclear	Unclear	Unclear	Low	Unclear	Intervention 284 (4.41 ± 4.38) Control 289 (4.39 ± 4.25)
Phelan 2014 [121]	USA	6-month 12-month	pregnancy	>18	Women	401	behavioral intervention	1-Depression 2-Stress	Higher scorer indicating more mental problems	1-Edinburgh Postnatal Depression Scale 2-Perceived Stress Scale	Low	Unclear	Low	Low	Low	Unclear	Depression 6-month Intervention 128 (5.1 ± 4.2) Control 133 (4.4 ± 3.6) 12-month Intervention 128 (5.6 ± 4.2) Control 133 (4.9 ± 4.1) Stress 6-month Intervention 128 (8.3 ± 3.0) Control 133 (7.8 ± 2.9) 12-month Intervention 128 (8.4 ± 2.8) Control 133 (8.1 ± 2.9)
Psarraki 2021 [122]	Greece	Unknown	major depressive disorder	18–65	83.9% women	69	Pythagorean Self-Awareness	1-Depression 2-Anxiety 3-Stress	Higher scorer indicating more mental problems	1-Depression anxiety and stress scale 2-Beck Depression Inventory	Low	Unclear	High	High	Unclear	Unclear	Depression Intervention 30 (13.31 ± 9.71) Control 32 (18.41 ± 12.66) Anxiety Intervention 30 (14.77 ± 11.07) Control 32 (15.19 ± 12.59) Stress Intervention 30 (14.89 ± 9.69) Control 32 (19.40 ± 10.08) BDI Intervention 30 (14.70 ± 9.77) Control 32 (22.28 ± 13.45)
Sacco 2009 [123]	USA	6-month	type 2 diabetes	18–65	Both %Women is unknown	62	regular telephone intervention	1-Depression	Higher scorer indicating more mental problems	1-Patient Health Questionnaire	High	Unclear	Unclear	Unclean	Low	Unclear	Intervention 31 (14.74 ± 5.96) Control 31 (16.87 ± 7.39)
Sanaati 2017 [124]	Iran	8-week	Pregnancy	27.5 (4.9) in intervention 27.7 (4.9) in control	Women	125	lifestyle-based education	1-Depression 2-Anxiety	Higher scorer indicating more mental problems	1-Edinburgh Postnatal Depression Scale 2-Spielberger State-Trait Anxiety Inventory	Low	Low	High	Low	Low	Unclear	Depression Intervention 62 (4.6 ± 3.5) Control 63 (7.5 ± 3.7) State anxiety Intervention 62 (34.4 ± 6.4) Control 63 (39.1 ± 9.2) Trait anxiety Intervention 62 (33.4 ± 7.1) Control 63 (39.0 ± 8.3)
Saxton 2014 [125]	UK	6-month	breast cancer	55.8 (10.0) in intervention 55.3 (8.8) in control	Women	85	pragmatic lifestyle intervention	1-Depression 2-Stress	Higher scorer indicating more mental problems	1-Beck Depression Inventory 2-Perceived Stress Scale	Low	Low	Unclear	Low	Low	Unclear	Depression Intervention 44 (5.1 ± 4.9) Control 41 (7.9 ± 6.0) Stress Intervention 44 (18.2 ± 7.7) Control 41 (19.5 ± 6.8)
Sebregts 2005 [126]	the Netherlands	Post-intervention 9-month	acute myocardial infarction or coronary artery bypass grafting	55.6 [8.0 in intervention 55.2 [9.7] in control	Both %Women is unknown	184	hort behavior modification program	1-Depression	Higher scorer indicating more mental problems	1-Beck Depression Inventory	Unclean	Low	Low	Unclear	Low	Unclear	Post-intervention Intervention 83 (7.7 ± 6.0) Control 75 (5.8 ± 4.9) 9-month Intervention 83 (6.9 ± 4.8) Control 75 (5.8 ± 5.1)
Serrano Ripoll 2015 [127]	Spain	6-month 12-month	Primary Care patients	IQR 40–61	82%women	273	Lifestyle change recommendations	1-Depression 2-Anxiety	Higher scorer indicating more mental problems	1-Beck Depression Inventory 2-Spielberger State-Trait Anxiety Inventory	Unclear	Low	Low	Low	Low	Unclear	Depression 6-month Intervention 106 (18.2 ± 9.98) Control 120 (16.6 ± 12.57) 12-month Intervention 95 (17.3 ± 9.44) Control 99 (16.1 ± 11.42) Anxiety 6-month Intervention 106 (70.9 ± 25.47) Control 120 (62.3 ± 27.10) 12-month Intervention 95 (67.8 ± 20.88) Control 99 (61.0 ± 29.95)
Sheean 2021 [128]	USA	12-week	metastatic breast cancer	≥18	Women	35	lifestyle intervention	1-Depression 2-Anxiety 3-Stress	Higher scorer indicating more mental problems	1-Hospital anxiety and depression scale 2-Perceived Stress Scale	Low	Low	Unclear	Low	Unclear	Unclear	Depression Intervention 17 (3.2 ± 3.1) Control 18 (3.4 ± 3.6) Anxiety Intervention 17 (5.8 ± 3.9) Control 18 (4.6 ± 5.1) Stress Intervention 17 (14.0 ± 6.2) Control 18 (12.3 ± 9.4)
Sorensen 1999 [129]	Norway	Unknown	elevated risk factors for cardiovascular disease	41–50	Both %Women is unknown	219	exercise and diet	1-Depression 2-Anxiety	Higher scorer indicating more mental problems	1-General Health Questionnaire 2- Symptom Check List-90 (SCL-90)	Unclear	Unclear	Unclear	Unclear	Unclear	Unclear	Depression Intervention 67 (2.0 ± 1.7) Control 43 (2.7 ± 2.9) Anxiety Intervention 67 (4.8 ± 5.2) Control 43 (7.5 ± 6.8)
Speyer 2016 [130]	Denmark	Unknown	Schizophreni/abdominal obesity	38.6 ± 12.4	56.1 women	428	lifestyle coaching	1-Stress	Higher scorer indicating more mental problems	2-Perceived Stress Scale	Low	Low	Low	Low	Low	Unclear	Intervention 138 (26.8 ± 7.8) Control 148 (25.5 ± 7.4)
Sylvia 2019 [131]	USA	20-week	bipolar disorder	18–65	65.8% women	38	Nutrition exercise wellness treatment	1-Depression	Higher scorer indicating more mental problems	1-Montgomery Asberg Depression Rating Scale 2-Clinical Global Impression Scale	Unclear	Unclear	Unclear	Low	Low	Unclear	CGI Intervention 19 (2.3 ± 0.9) Control 19 (2.4 ± 1.0) MADRS Intervention 19 (8.1 ± 6.7) Control 19 (9.6 ± 8.2)
Takeda 2020 [132]	Japan	7-month	Elderly	77.03 (8.08 in intervention 75.51 (6.55) in control	88.2% women	127	lifestyle development program	1-Depression	Higher scorer indicating more mental problems	1–15-item Geriatric Depression Scale	Unclear	Unclear	Unclear	Unclear	Unclear	Unclear	Intervention 60 (4.20 ± 2.93) Control 67 (4.64 ± 3.61)
Toobert 2007 [133]	USA	6-month 12-month 24-month	type 2 diabetes	61.1 (8.0) in intervention 60.7 (7.8) in control	Women	279	Mediterranean lifestyle program	1-Depression 2-Stress	Higher scorer indicating more mental problems	1-Center for Epidemiologic Studies Depression Scale 2-Perceived Stress Scale	Unclear	Unclear	Unclear	Unclear	Low	Unclear	Depression 6-month Intervention 163 (13 ± 11) Control 116 (15 ± 12) 12-month Intervention 163 (15 ± 11) Control 116 (14 ± 9) 24-month Intervention 163 (12 ± 11) Control 116 (14 ± 10) Stress 6-month Intervention 163 (2.5 ± 0.62) Control 116 (2.6 ± 0.59) 12-month Intervention 163 (2.6 ± 0.66) Control 116 (2.6 ± 0.58) 24-month Intervention 163 (2.4 ± 0.64) Control 116 (2.6 ± 0.61)
Tousman 2011 [134]	USA	2-month	Asthma	51.4 (14.7) in intervention 55.0 (10.0)	68.9% women	45	behavior modification procedure	1-Depression	Higher scorer indicating more mental problems	1-Geriatric Depression Scale	Unclear	Unclear	Unclear	Unclear	Unclear	Unclear	Intervention 21 (1.8 ± 2.1) Control 24 (1.9 ± 2.1)
Trento 2020 [135]	Italy	4-year	type 2 diabetes	62.6 ± 7.5 in intervention ± 9.1 in control	36% women	50	Self-management education	1-Depression 2-Anxiety	Higher scorer indicating more mental problems	1-Hospital anxiety and depression scale	Unclean	Low	High	High	Low	Unclear	Depression Intervention 24 (4.5 ± 3.86) Control 25 (3.44 ± 2.95) Anxiety Intervention 24 (4.83 ± 3.25) Control 25 (5.28 ± 3.45)
Tsai 2021 [136]	Taiwan	2-week	at-risk mental state	20–35	55.4% women	92	Health-Awareness-Strengthening Lifestyle	1-Anxiety	Higher scorer indicating more mental problems	1-State and Trait Anxiety Inventory	Low	Unclear	Unclear	Unclear	Low	Unclear	State Anxiety Intervention 46 (42.5 ± 7.7) Control 46 (47.7 ± 9.5) Trait Anxiety Intervention 46 (52.0 ± 7.0) Control 46 (56.0 ± 7.0)
Ural 2021 [137]	Turkey	6-week	gestational diabetes mellitus	≥18	Women	88	health-promoting lifestyle education	1-Depression	Higher scorer indicating more mental problems	1-Center for Epidemiologic Studies Depression Scale	High	High	Unclear	Unclear	Unclear	Unclear	Intervention 46 (14.93 ± 9.39) Control 42 (15.74 ± 8.54)
Van Dammen 2019 [138]	the Netherlands	5-year	Obesity and infertility	18–39	Women	577	lifestyle intervention	1-Depression 2-Anxiety 3-Stress	Higher scorer indicating more mental problems	1-Hospital anxiety and depression scale 2-Perceived Stress Scale-10	Unclear	Unclear	Unclear	Unclear	Unclear	Unclear	Depression Intervention 84 (7.7 ± 3.66) Control 94 (7.7 ± 2.90) Anxiety Intervention 84 (8.0 ± 3.66) Control 94 (8.2 ± 2.90) Stress Intervention 52 (14.4 ± 6.48) Control 63 (13.7 ± 4.76)
van der Wulp 2012 [139]	the Netherlands	3-month 6-month	type 2 diabetes	Unknown	45.4% women	119	peer-led self-management	1-Depression	Higher scorer indicating more mental problems	1-Center for Epidemiologic Studies Depression Scale	Unclear	Unclear	Unclear	Unclear	Low	Unclear	3-month Intervention 59 (10.60 ± 8.03) Control 60 (11.20 ± 8.52) 6-month Intervention 59 (8.64 ± 8.56) Control 60 (12.07 ± 9.55)
Wang 2014 [140]	USA	4-month 12-month	type 2 diabetes	≥18	76.6 women	252	Diabetes Self-Management	1-Depression	Higher scorer indicating more mental problems	1-Center for Epidemiologic Studies Depression Scale	Low	Low	Unclear	Low	Unclear	Unclear	4-month Intervention 117 (17.5 ± 13.0) Control 112 (21.8 ± 12.4) 12-month Intervention 109 (18.5 ± 13.0) Control 107 (22.6 ± 13.4)
Wang 2017 [141]	China	1-month 3-month	Metabolic syndrome	24–78	50.9% women	173	lifestyle intervention program	1-Depression	Higher scorer indicating more mental problems	1-Hospital anxiety and depression scale	Low	Low	Unclear	Low	Low	Unclear	1-month Intervention 86 (3.23 ± 2.71) Control 87 (3.94 ± 3.49) 3-month Intervention 86 (2.13 ± 2.06) Control 87 (3.43 ± 2.96)
Williams 2018 [142]	Australia	26-week	Low Back Pain	56.7 ± 13.4	59.1% women	159	Healthy Lifestyle Intervention	1-Depression 2-Anxiety 3-Stress	Higher scorer indicating more mental problems	1-Depression anxiety and stress scale	Low	Low	Unclear	Low	Low	Low	Depression Intervention 43 (13.1 ± 11.2) Control 61 (11.9 ± 11.1) Anxiety Intervention 43 (9.8 ± 8.3) Control 61 (9.4 ± 9.0) Stress Intervention 43 (14.3 ± 10.7) Control 61 (13.8 ± 11.1)
Wong 2021 [143]	China	9-week	moderate level of depressive symptoms	32.9 ± 12.5	84.8% women	79	Lifestyle Medicine	1-Depression 2-Anxiety	Higher scorer indicating more mental problems	1-Patient Health Questionnaire 2-General Anxiety Disorder	Low	Unclear	High	Unclean	Unclear	Unclear	Depression Intervention 39 (8.8 ± 3.8) Control 40 (11.6 ± 4.7) Anxiety Intervention 39 (7.8 ± 3.2) Control 40 (11.5 ± 4.6)
Sample size and *p* value																	
Advocat 2016 [65]	Australia	6-month	Parkinson’s disease	18–75	57.9% women	48	Mindfulness-based lifestyle	1-Depression 2-Anxiety 3-Stress	Higher scorer indicating more mental problems	1-Depression anxiety and stress scale	Low	Low	Low	Low	High	Unclear	Depression Intervention 23 Control 25 *p* = 0.54 Anxiety Intervention 23 Control 25 *p* = 0.38 Stress Intervention 23 Control 25 *p* = 0.04
Brown 2006 [71]	UK	6-week	Serious mental illness	18–65	85.7% women	17	health promotion sessions	1-Depression 2-Anxiety	Higher scorer indicating more mental problems	1-Hospital anxiety and depression scale	Unclear	Low	Unclear	Low	Low	Unclear	Depression Intervention 7 Control 10 *p* = 0.080 Anxiety Intervention 7 Control 10 *p* = 0.190
Gallagher 2014 [144]	Australia	16-week	overweight with heart disease and diabetes	63.2 ± 8.69	40% women	147	Group-based lifestyle intervention	1-Depression	Higher scorer indicating more mental problems	1-Hospital anxiety and depression scale	Low	Unclear	Unclear	Unclear	Low	Unclear	Intervention 75 Control 58 *p* = 0.014
Gaudel 2021 [145]	Nepal	1-month	coronary artery disease	>18	24.1% women	224	lifestyle-related risk factor modification intervention	1-Stress	Higher scorer indicating more mental problems	1-Perceived Stress Scale-10	Low	Unclear	High	Unclear	Unclear	Unclear	Intervention 98 Control 98 *p* = 0.000
Goracci 2016 [146]	Italy	12-month	Recurrent depression	>18	80% women	160	lifestyle intervention	1-Depression	Higher scorer indicating more mental problems	1-Patient Health Questionnaire	Unclear	Unclear	Unclear	Unclear	Low	Unclear	Intervention 81 Control 79 *p* = 0.29
Islam 2013 [97]	USA	6-month	at risk for diabetes	18–75	64.3% women	35	Healthy Lifestyles	1-Depression 2-Anxiety	Higher scorer indicating more mental problems	1-Patient Health Questionnaire 2-Generalized Anxiety Disorder Scale	Unclear	Unclear	Unclear	Unclear	Unclear	Unclear	Depression Intervention 21 Control 14 *p* = 0.43 Anxiety Intervention 21 Control 14 *p* = 0.15
Jiskoot 2020 [147]	The Netherlands	12-month	Polycystic Ovary Syndrome	18–38	Women	120	lifestyle intervention	1-Depression	Higher scorer indicating more mental problems	1-Beck Depression Inventory-II	Low	Unclear	Unclear	Unclear	Low	Unclear	Intervention 60 Control 60 *p* = 0.045
Jørstad 2016 [99]	the Netherlands	12-month	Acute coronary syndrome	18–80	22.5% women	120	nurse-coordinated prevention programme	1-Depression	Higher scorer indicating more mental problems	1-Beck Depression Inventory-II	Low	Unclear	Unclear	Unclear	Unclear	Unclear	Intervention 54 Control 66 *p* = 0.03
Kokka 2019 [104]	Greece	8-week	Intimate Partner Violence	18–70	Women	60	stress management program	1-Depression 2-Anxiety 3-Stress	Higher scorer indicating more mental problems	1-Depression anxiety and stress scale	Low	Unclean	High	High	Low	Low	Depression Intervention 30 Control 30 *p* = 0.000 Anxiety Intervention 30 Control 30 *p* = 0.000 Stress Intervention 30 Control 30 *p* = 0.000
Lorig 2009 [148]	USA	6-month	type 2 diabetes	24–93	Both %Women is unknown	294	Peer-Led Diabetes Self-management	1-Depression	Higher scorer indicating more mental problems	1-Patient Health Questionnaire	Low	Unclean	High	Unclean	Low	Unclear	Intervention 161 Control 133 *p* = 0.000
Lovell 2014 [149]	UK	6-month 12-month	psychosis	16–35	40% women	105	Healthy Living Intervention	1-Depression	Higher scorer indicating more mental problems	1-Calgary Depression Scale.	Low	Unclean	High	Low	Low	Unclear	6-month Intervention 46 Control 39 *p* = 0.98 12-month Intervention 48 Control 42 *p* = 0.65
Mitchell 2014 [160]	UK	6-month	chronic obstructive pulmonary disease	69 ± 8.0 in intervention 69 ± 10.1 in control	45.1% women	184	self-management programme	1-Depression 2-Anxiety	Higher scorer indicating more mental problems	1-Hospital anxiety and depression scale	Low	Low	High	Low	Low	Unclear	Depression Intervention 89 Control 95 *p* = 0.27 Anxiety Intervention 89 Control 95 *p* = 0.04
Pelekasis 2016 [150]	Greece	8-week	Breast Cancer	18–75	Women	61	stress management	1-Depression 2-Anxiety 3-Stress	Higher scorer indicating more mental problems	1-Depression anxiety and stress scale	Low	Unclear	Unclear	Unclear	Low	Unclear	Depression Intervention 25 Control 28 *p* = 0.01 Anxiety Intervention 25 Control 28 *p* = 0.005 Stress Intervention 25 Control 28 *p* = 0.002
Przybylko 2021 [151]	Australia and New Zealand	12-week 24-week	General population	46.97 ± 14.5	69.9% women	320	Online interdisciplinary intervention	1-Depression 2-Anxiety 3-Stress	Higher scorer indicating more mental problems	1-Depression anxiety and stress scale	Low	Unclean	High	High	Low	Unclear	Depression 12-week Intervention 159 Control 162 *p* = 0.002 24-week Intervention 159 Control 162 *p* = 0.005 Anxiety 12-week Intervention 159 Control 162 *p* = 0.000 24-week Intervention 159 Control 162 *p* = 0.035 Stress 12-week Intervention 159 Control 162 *p* = 0.001 24-week Intervention 159 Control 162 *p* = 0.000
Rosal 2005 [152]	USA	3-month 6-month	type 2 diabetes	45–82	80% women	25	Diabetes Self-Management	1-Depression	Higher scorer indicating more mental problems	1- Center for Epidemiologic Studies Depression Scale	Unclear	Unclear	Unclear	Unclear	Unclear	Unclear	3-month Intervention 15 Control 10 *p* < 0.05 6-month Intervention 15 Control 10 *p* = 0.01
Ruusunen 2012 [153]	Finland	Unknown	overweight or obese/glucose tolerance	40–64	57.9% women	140	lifestyle intervention	1-Depression	Higher scorer indicating more mental problems	1-Beck Depression Inventory	Unclear	Unclear	Unclear	Unclear	Unclear	Unclear	Intervention 69 Control 71 *p* = 0.965
Samuel-hodge 2017 [154]	USA	20-week	overweight or obesity and type 2 diabetes	21–75	81% women	108	Lifestyle Support	1-Depression	Higher scorer indicating more mental problems	1-Patient Health Questionnaire	Unclear	Unclear	High	Low	Unclear	Unclear	Intervention 34 Control 16 *p* = 0.01
Skrinar 2005 [155]	USA	12-week	Serious Psychiatric Disabilities	18–55	Unknown	20	healthy lifestyle	1-Depression 2-Anxiety	Higher scorer indicating more mental problems	Symptom Check List-90 (SCL-90)	Unclear	Unclear	Unclear	Unclear	Unclear	Unclear	Depression Intervention 9 Control 11 *p* = 0.09 Anxiety Intervention 9 Control 11 *p* = 0.59
Surkan 2012 [156]	USA	Unknown	Postpartum	18–44	Women	403	Health Promotion Intervention	1-Depression	Higher scorer indicating more mental problems	1- Center for Epidemiologic Studies Depression Scale	Unclear	Unclear	Unclear	Unclear	Low	Unclear	Intervention 203 Control 200 *p* = 0.046
Ye 2016 [157]	China	2-month 6-month 12-month	Breast cancer	Unknown	Women	204	mentor-based program	1-Depression 2-Anxiety	Higher scorer indicating more mental problems	1-Hospital anxiety and depression scale	Unclear	Unclear	Unclear	Unclear	Unclear	Unclear	Depression 2-month Intervention 99 Control 101 *p* = 0.0019 6-month Intervention 95 Control 92 *p* = 0.000 12-month Intervention 90 Control 81 *p* = 0.000 Anxiety 2-month Intervention 100 Control 102 *p* = 0.0485 6-month Intervention 96 Control 91 *p* = 0.000 12-month Intervention 91 Control 79 *p* = 0.000

* calculated by author(s).

### 3.2. Quality Assessment of Studies

A qualitative evaluation of the eligible studies was conducted, based on the results of the qualitative evaluation listed in Table 1.

### 3.3. Lifestyle Intervention and Depression

A meta-analysis of 89 randomized clinical trials of lifestyle interventions on depression indicated that lifestyle interventions lead to a reduction in depression, according to which Hedges’s g was equal to −0.21 with 95% confidence interval −0.26, −0.15 (Z = −7.12; *p* < 0.001; *I*^2^ = 56.57) (not shown in the figure).

### 3.4. Sub-Group Analysis for Lifestyle Intervention and Depression

Table 2 shows the results of the meta-analysis of lifestyle interventions for depression across different populations. In the cancer population, lifestyle interventions led to a reduction in depression (Hedges’s g = −0.34; 95% CI −0.59, −0.08 [Z = −2.54; *p* = 0.011; *I*^2^ = 56.23%]). In the depressed population, lifestyle interventions led to a reduction in depression [Hedges’ g = −0.44; 95% CI −0.62, −0.26; Z = −4.82; *p* < 0.001; *I*^2^ = 40.46%). In the diabetes/at-risk diabetes population, lifestyle interventions led to a reduction in depression [Hedges’ g = −0.15; 95% CI −0.27, −0.03 (Z = −2.43; *p* = 0.015; *I*^2^ = 56.51%). In the heart-related disease population, lifestyle interventions led to a reduction in depression (Hedges’s g = −0.19; 95% CI −0.34, −0.04 [Z = −2.44; *p* = 0.015; *I*^2^ = 39.52%]). In the metabolic syndrome population, lifestyle interventions led to a reduction in depression [Hedges’ g = −0.74; 95% CI −1.27, −0.21 (Z = −2.44; *p* = 0.006; *I*^2^ = 69.66%). In obstructive pulmonary disease, older adults, and the overweight/obese population, lifestyle interventions did not affect depression significantly.

**Figure 2:**
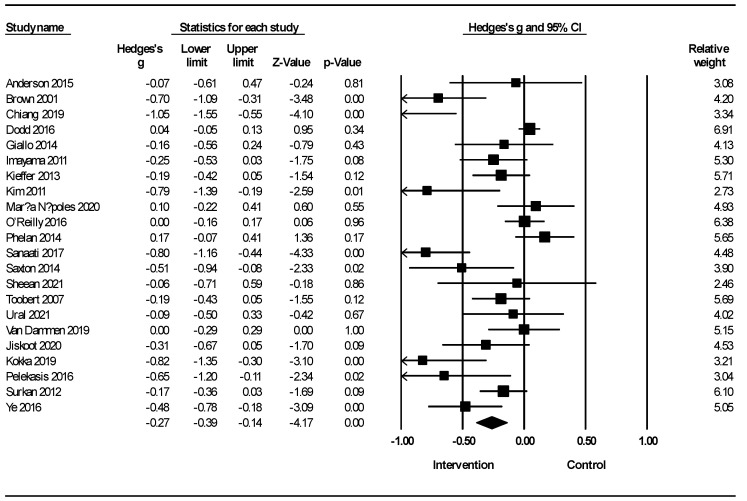
Forest plot for lifestyle intervention on depression in women [66,70,76,81,85,94,101,102,104,109,120,121,124,125,128,133,137,138,147,150,156,157].

Figure 2 shows the meta-analysis of lifestyle interventions on depression in women. In this case, lifestyle interventions led to a reduction in depression (Hedges’s g = −0.27; 95% CI −0.39, −0.14; Z = −4.17; *p* < 0.001; *I*^2^ = 75.25%). Owing to the insufficient number of studies, a similar meta-analysis for the male population could not be procured.

Table 3 shows the meta-analysis of lifestyle interventions on depression based on depression scales. Lifestyle interventions on depression in the Beck Depression Inventory (BDI) indicated a reduction in depression post-intervention [Hedges’ g = −0.26; 95% CI −0.45, −0.07; Z = −2.62; *p* = 0.009; *I*^2^ = 73.07%). Lifestyle interventions on depression in the Center for Epidemiologic Studies Depression Scale (CES-D) showed that lifestyle interventions led to a reduction in depression [Hedges’ g = −0.23; 95% CI −0.32, −0.14 (Z = −4.97; *p* < 0.001; *I*^2^ = 49.69%). Lifestyle interventions on depression in the Hospital Anxiety and Depression Scale (HADS) showed that lifestyle interventions led to a reduction in depression [Hedges’ g = −0.25; 95% CI −0.35, −0.14; Z = −4.62; *p* < 0.001; *I*^2^ = 30.95%). Lifestyle interventions on depression in the Patient Health Questionnaire (PHQ) showed that lifestyle interventions led to a reduction in depression [Hedges’s g = −0.16; 95% CI −0.28, −0.05 (Z = −2.76; *p* = 0.006; *I*^2^ = 49.46%).

### 3.5. Lifestyle Intervention and Anxiety

**Figure 3:**
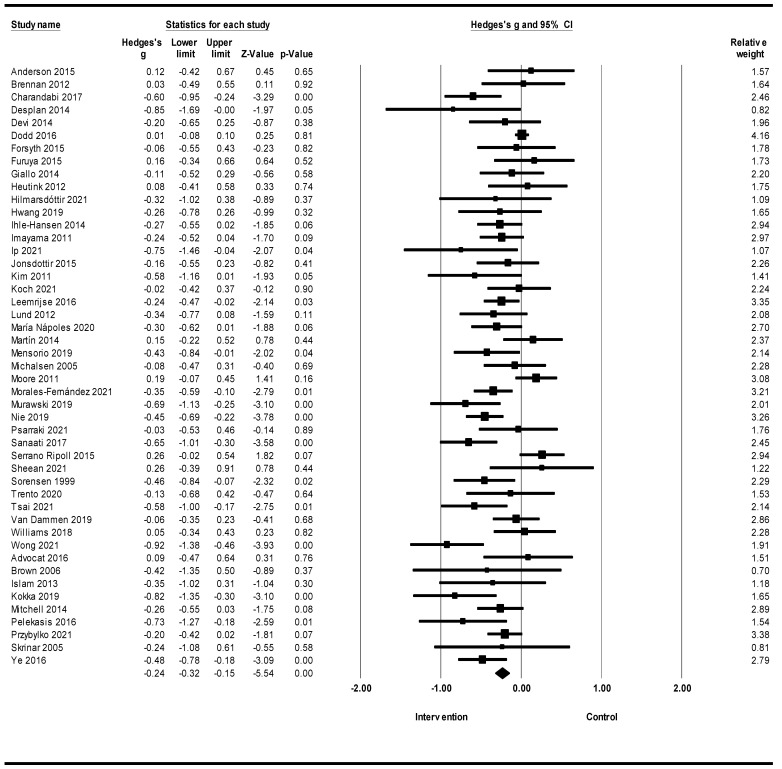
Forest plot of lifestyle intervention on anxiety [65,66,68,71,75,79,80,81,82,83,85,89,90,92,94,96,97,98,102,103,104,107,108,109,110,112,113,117,118,122,124,127,128,129,135,136,138,142,143,150,151,155,157,158,159,160].

A meta-analysis of 47 randomized clinical trials of lifestyle interventions on anxiety showed that lifestyle interventions led to a reduction in anxiety, according to which Hedges’s g was equal to −0.24 with a 95% confidence interval of −0.32, −0.15 (Z = −5.54; *p* < 0.001; *I*^2^ = 59.25) (Figure 3).

### 3.6. Sub-Group Analysis Lifestyle Intervention and Anxiety

**Figure 4:**
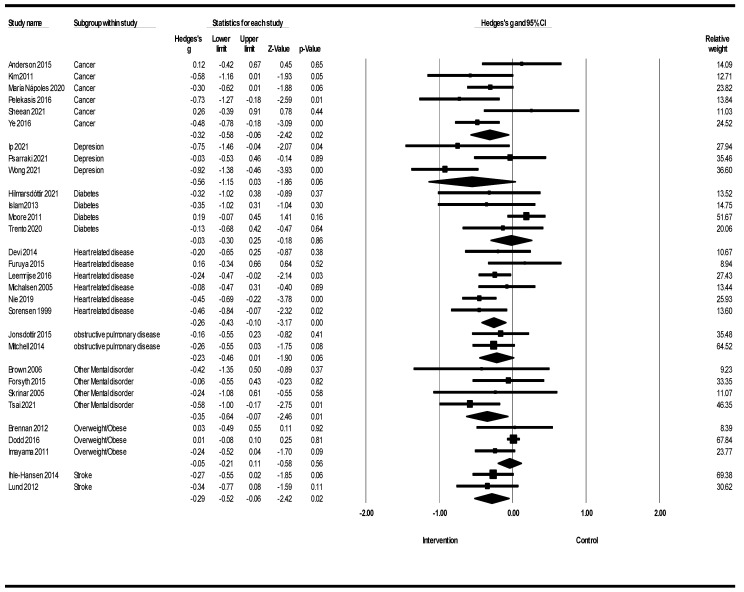
Forest plot for lifestyle intervention on anxiety based on diseases [66,68,71,80,81,82,83,90,93,94,96,97,98,102,107,108,109,113,118,122,128,129,135,136,143,150,155,157,158,160].

Figure 4 shows the meta-analysis of lifestyle interventions for anxiety based on different populations. Lifestyle interventions on anxiety in the cancer population showed that they led to a reduction in anxiety (Hedges’s g = −0.32; 95% CI −0.58, −0.06; Z = −2.42; *p* = 0.015; *I*^2^ = 47.10%). Lifestyle interventions for anxiety in the heart-related disease population showed that lifestyle interventions led to a reduction in anxiety [Hedges’s g = −0.26; 95% CI −0.43, −0.10; Z = −3.17; *p* = 0.002; *I*^2^ = 30.52%]. Lifestyle interventions on anxiety in other mental disorder populations showed that they led to a reduction in anxiety (Hedges’s g = −0.35; 95% CI −0.64, −0.07; Z = −2.46; *p* = 0.014; *I*^2^ = 0%). Lifestyle interventions on anxiety in the stroke population reduced anxiety (Hedges’s g = −0.29; 95% CI −0.52, −0.06; Z = −2.42; *p* = 0.015; *I*^2^ = 0%). Lifestyle interventions for anxiety in depressed, diabetic, overweight/obese, and obstructive pulmonary disease populations showed that their effects on anxiety were not significant.

**Figure 5:**
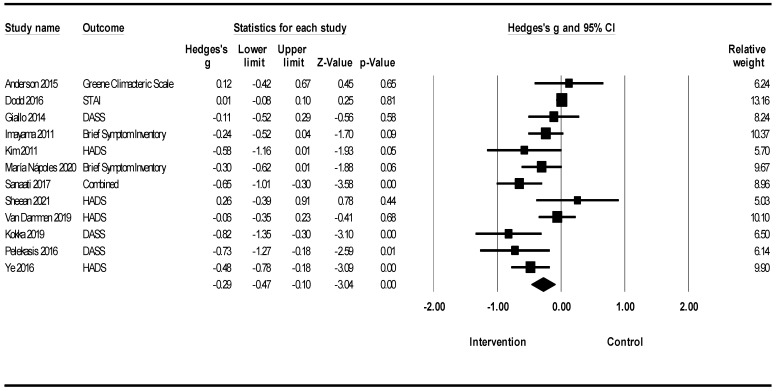
Forest plot for lifestyle intervention on anxiety in women [66,81,85,94,102,104,109,124,128,138,150,157].

Figure 5 shows the meta-analysis of lifestyle interventions for anxiety among women. Lifestyle interventions for anxiety in women led to reduced anxiety (Hedges’ g = −0.29; 95% CI −0.47, −0.10; Z = −3.04; *p* = 0.002; *I*^2^ = 72.86%). Owing to the insufficient number of studies, a similar meta-analysis for the male population could not be accomplished.

**Figure 6:**
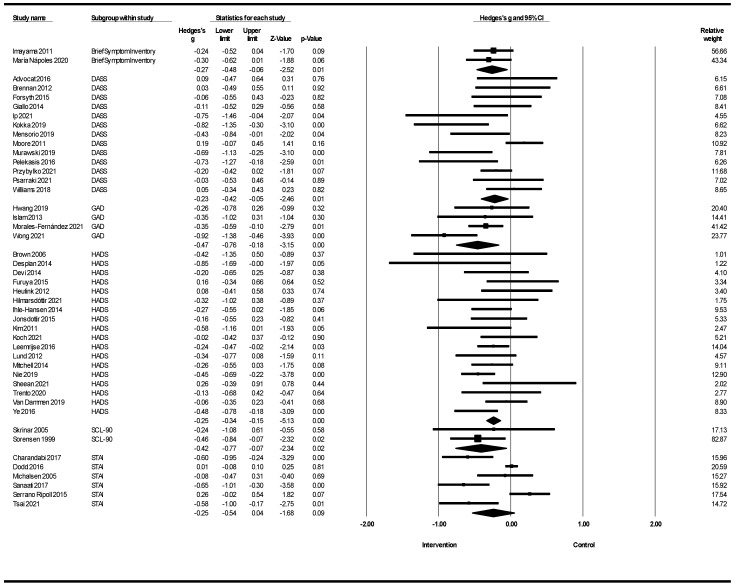
Forest plot for lifestyle intervention on anxiety based on anxiety scales [65,68,71,75,79,80,81,82,83,85,89,90,92,93,94,97,98,102,103,104,107,108,109,112,117,118,122,127,128,129,135,136,138,143,150,155,157,158,159,160].

Figure 6 shows a meta-analysis of lifestyle interventions for anxiety based on anxiety scales. Lifestyle interventions on anxiety in the Brief Symptom Inventory (BSI) showed that lifestyle interventions led to a reduction in anxiety (Hedges’s g = −0.27; 95% CI −0.48, −0.06; Z = −2.52; *p* = 0.012; *I*^2^ = 0%). Lifestyle interventions for anxiety in the DASS showed a reduction in anxiety [Hedges’ g = −0.23; 95% CI −0.42, −0.05 (Z = −2.46; *p* = 0.014; *I*^2^ = 59.86%). Lifestyle interventions on anxiety in Generalized anxiety disorder (GAD) showed a reduction in anxiety (Hedges’s g = −0.47; 95% CI −0.76, −0.18; Z = −3.15; *p* = 0.000; I2 = 43.36%). Lifestyle interventions for anxiety in the HADS showed a reduction in anxiety (Hedges’ g = −0.25; 95% CI −0.34, −0.15; Z = −5.13; *p* = 0.001; *I*^2^ = 8.37%). Lifestyle interventions on anxiety in the SCL−90 showed a reduction in anxiety (Hedges’s g = −0.42; 95% CI −0.77, −0.07; Z = −2.34; *p* = 0.019; *I*^2^ = 0%). Lifestyle interventions for anxiety were not significant in the STAI group.

### 3.7. Lifestyle Intervention and Stress

**Figure 7:**
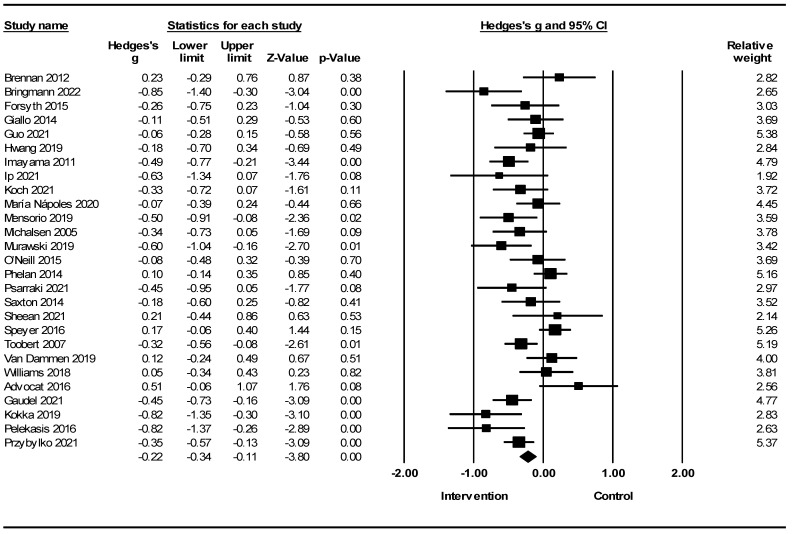
Forest plot for lifestyle intervention on stress [65,68,69,82,85,87,92,94,96,103,104,109,112,113,117,119,121,122,125,128,130,133,142,145,150,151,161].

A meta-analysis of 27 randomized clinical trials of lifestyle interventions on stress showed a reduction in stress, according to which Hedges’ g was equal to −0.22 with a 95% confidence interval −0.34, −0.11 (Z = −3.80; *p* < 0.001; *I*^2^ = 61.40) (Figure 7).

**Figure 8:**
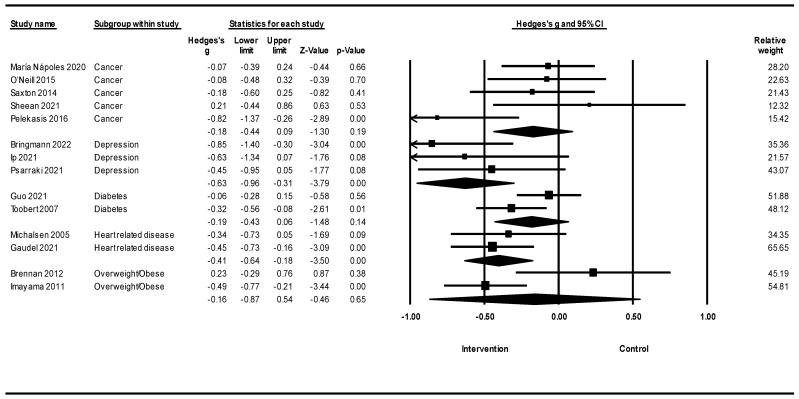
Forest plot for lifestyle intervention on stress based on diseases [68,69,82,94,96,113,119,122,125,128,133,145,150].

Figure 8 shows a meta-analysis of lifestyle interventions on stress based on different populations. Lifestyle interventions for stress in depressed populations showed a reduction in stress [Hedges’ g = −0.63; 95% CI −0.96, −0.31; Z = −3.79; *p* < 0.001; *I*^2^ = 0%). Lifestyle interventions for stress in the heart-related disease population showed a reduction in stress [Hedges’s g = −0.41; 95% CI −0.64, −0.18; Z = −3.50; *p* < 0.001; *I*^2^ = 0%). Lifestyle interventions for stress in cancer, diabetes, and overweight/obese populations showed that the effects of lifestyle interventions on stress were not significant.

**Figure 9:**
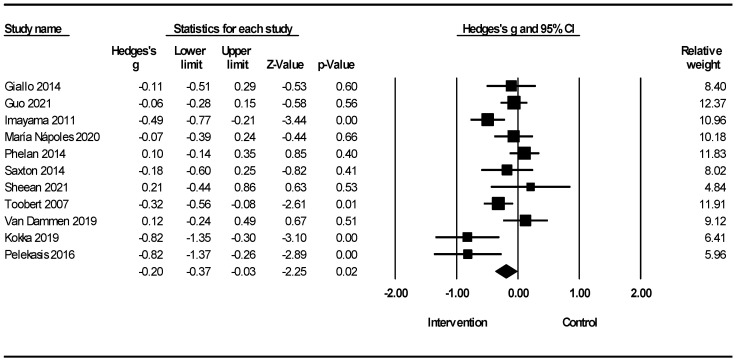
Forest plot for lifestyle intervention on stress in women [85,87,94,103,109,121,125,128,133,138,150].

Figure 9 shows a meta-analysis of lifestyle interventions for stress in women. Lifestyle interventions on stress in women showed a reduction in stress [Hedges’ g = −0.20; 95% CI −0.37, −0.03 (Z = −2.25; *p* = 0.024; *I*^2^ = 64.27%). Owing to the insufficient number of studies, a similar meta-analysis on the male population could not be performed.

**Figure 10:**
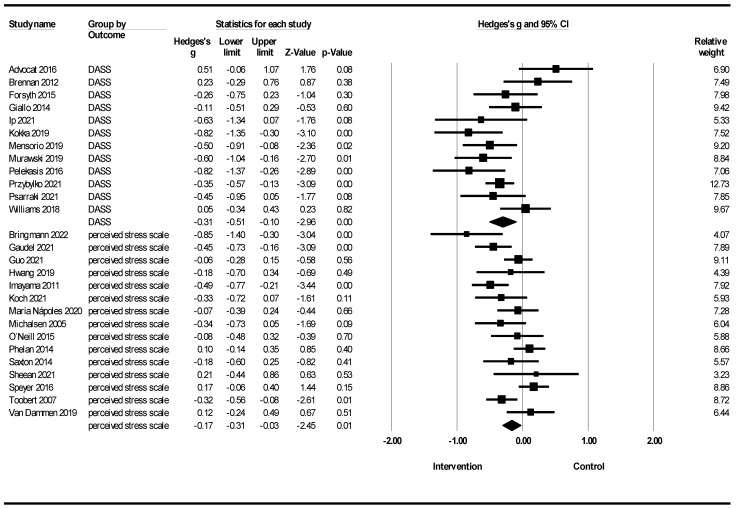
Forest plot for lifestyle intervention on stress based on stress scales [65,68,69,82,85,87,94,96,103,109,112,113,117,119,121,122,125,128,130,133,138,142,144,145,147,150,151].

Figure 10 shows the meta-analysis of lifestyle interventions for stress based on the stress scales. Lifestyle interventions for stress in the DASS showed a reduction in stress [Hedges’ g = −0.31; 95% CI −0.51, −0.10; Z = −2.96 2; *p* = 0.003; *I*^2^ = 59.42%). Lifestyle interventions on stress in the Perceived Stress Scale (PSS) showed a reduction in stress (Hedges’ g = −0.17; 95% CI −0.31, −0.03; Z = −2.45; *p* = 0.014; *I*^2^ = 60.77%]).

### 3.8. Publication Bias and Heterogeneity

**Figure 11:**
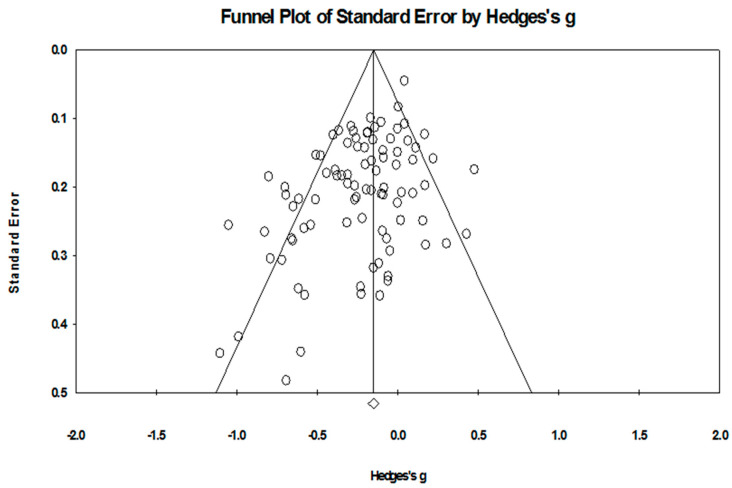
Funnel plot for lifestyle intervention and depression.

In a meta-analysis of lifestyle interventions on depression, the Q test showed 202.62 (d.f. 88; *p* < 0.001), and *I*^2^ was 56.57%, and showed moderate heterogeneity [59]. The funnel plot in Figure 11 showed that there is a publication bias. Egger’s test indicated *p* < 0.001 and showed publication bias. The trim-and-fill imputed 14 studies, and the adjusted Hedges’ g was equal to −0.14 with 95% confidence intervals −0.20, −0.08 [64].

**Figure 12:**
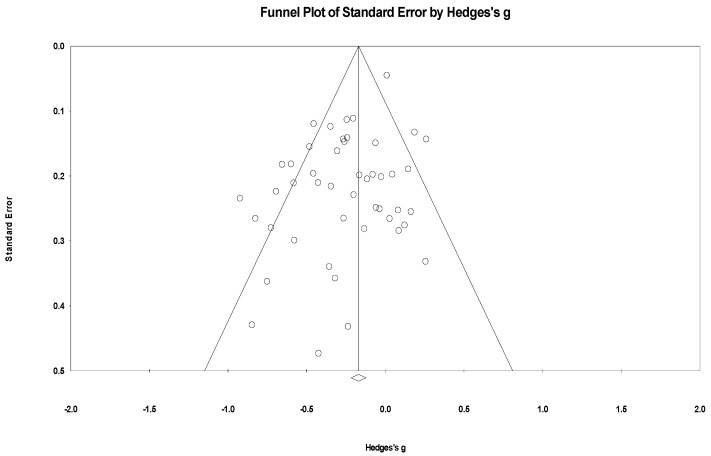
Funnel plot for lifestyle intervention and anxiety.

In a meta-analysis of lifestyle interventions on anxiety, the Q test showed 112.89 (d.f 47; *p* < 0.001), and *I*^2^ was 59.25%, and showed moderate heterogeneity [59]. The funnel plot in Figure 12 indicates the publication bias. Egger’s test was *p* = 0.002 and showed publication bias; the trim-and-fill imputed four studies, and Hedges’ g was equal to −0.20 with a 95% confidence interval of −0.29, −0.12 [64].

**Figure 13:**
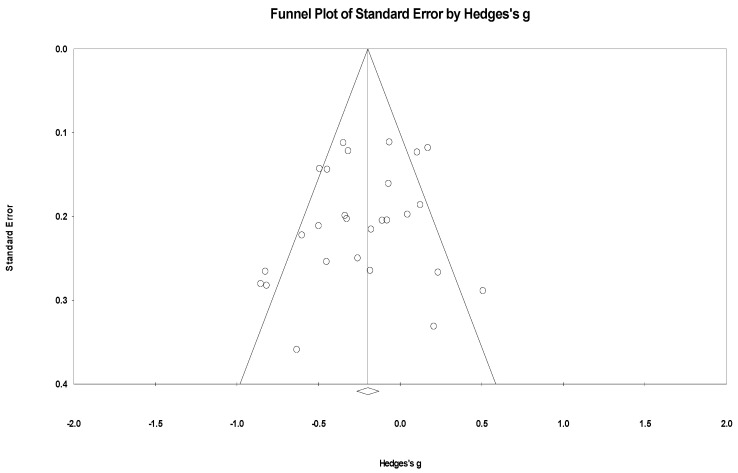
Funnel plot for lifestyle intervention and stress.

In a meta-analysis of lifestyle interventions on stress, the Q test showed 67.27 (d.f 26; *p* < 0.001), and *I*^2^ was 61.40%, and showed substantial heterogeneity [59]. The funnel plot in Figure 13 shows no publication bias. The Egger’s test scored *p* = 0.139 and did not show publication bias; the trim-and-fill test [64] did not impute any study.

## 4. Discussion

This systematic review and meta-analysis investigated the effects of lifestyle interventions on depression, anxiety, and stress in randomized clinical trials. This study included 96 eligible clinical trials to address research gaps noted in previous meta-analyses.

The results showed that lifestyle interventions led to improvements in depression, anxiety, and stress levels. This means that as people adopt a healthy lifestyle, their mental health improves. The finding related to the effect of lifestyle intervention on depression and anxiety is consistent with studies that have shown this effect [39,40], with the difference that the scope of the current study was much wider, and it was also methodologically strong because previous meta-analysis studies sometimes included clinical trials without a control group, or they combined an individual randomized clinical trial with a cluster. They also used non-parametric statistics, which can reduce the accuracy of the results, and all these factors can lead to weakness. A previous meta-analysis also showed a large effect size for the effect of lifestyle interventions on anxiety; however, that study was limited by the small number of studies included in the meta-analysis and the study population of overweight and obese women [161]. Unlike these previous analyses, our study systematically reviewed and meta-analyzed the impact of stress, which is a novel contribution to this field.

Our findings revealed that lifestyle interventions significantly reduced stress, with pronounced effects in individuals with depression, heart disease, and in women. The considerable role of stress in overall health has driven researchers to explore stress reduction methods over the past decade [162,163,164]. The results showed that lifestyle changes, such as exercise, diet, and improved sleep quality, can effectively reduce stress by lowering cortisol and increasing endorphin levels. Furthermore, the findings suggest that psychological factors, such as increased mindfulness and interoceptive awareness, may mediate these benefits [162,165]. Furthermore, stress and sleep quality are interrelated, and each affects the other in a bidirectional manner [166,167]. Moreover, sleep quality affects stress, and is also affected by stress, forming a vicious loop [168]. Similarly, while a healthy diet seems to reduce stress levels, higher levels of stress have been found to negatively impact diet quality [169]. In such cases, where a causes b, but also b causes a, it is important to target elements of the cycle that can be easier to break, which in such cases may be lifestyle changes rather than stress reductions. Additionally, among the three variables explored in this study (stress, depression, and anxiety), the effect size for lifestyle interventions was the highest for stress, suggesting a more pronounced effect. This further indicates the significance of the findings presented in this study, as stress has a direct impact on human health and influences epigenetic regulation [170]. Despite these negative effects, greater public awareness is required to highlight these direct links [171].

While previous reviews analyzing lifestyle interventions and depression have reported small and moderate effect sizes [43,172], the current review adds to the literature by confirming a modest effect size. In the current study, the effect of lifestyle interventions on depression was significant for individuals with depression, heart-related diseases, diabetes/at-risk diabetes, cancer, and metabolic syndrome, and for women. Interventions, such as healthy eating, increased physical activity, and exercise, have been found to have positive effects. A recent systematic review concluded that even low amounts of physical activity in a week can reduce the risk of developing depression by up to 18% compared to no activity [173].

Interventions based on a healthy lifestyle can affect mental health and reduce depression, anxiety, and stress through several mechanisms. One mechanism for the impact of lifestyle interventions on depression, anxiety, and stress involves neural mechanisms [174]. Physiological factors mediating the effects of physical activity and depression have been well studied, with findings that the effects of physical activity (as a lifestyle component) and antidepressant drugs on the relief of depression can occur through common neuro-molecular mechanisms [175,176] by increasing serotonin and norepinephrine, regulating the hypothalamus–pituitary–adrenal axis, and reducing systemic inflammatory signaling [177,178,179,180]. For anxiety and stress relief, studies have also shown similar neural mechanisms [181,182,183]. which helps reduce anxiety and stress. Healthy nutrition is another lifestyle mechanism that improves mental health [184]. For example, eating foods rich in carbohydrates can lead to diabetes and obesity [185] and. as studies have widely shown, obesity and diabetes are two important risk factors for depression, anxiety, and stress and lead to the deterioration of mental health [24,25,186,187,188,189]. Physiological mediating factors have also been explored to understand the role of a healthy diet in depression and overall affect, with some indications that the microbiome–gut–brain axis may be at its heart [187].

Anxiety: Similar to the findings regarding stress and depression, this study also found that physical activity, nutrition, and psychoeducation improved anxiety. The association between lifestyle interventions and reduced anxiety was prominent among patients with cancer, heart-related diseases, mental disorders, and women. With regard to the effect of physical activity on anxiety and stress relief, studies have also shown similar neural mechanisms [170,171,172], which help reduce anxiety and stress, as reported above. A healthy diet can positively affect anxiety through various mechanisms. These include the role of antioxidants, omega-3 fatty acids, zinc, probiotics, magnesium, and selenium in reducing the symptoms of anxiety disorders (citation). In the case of insufficient antioxidants, for example, oxidative stress has been linked to anxiety through pathways such as alterations in neurotransmission and neuronal function (citation). Moreover, an unhealthy diet can cause depression and anxiety by increasing blood glucose and glycemic load. It has been shown in animal studies that this concentration of high dietary glycemic load “leads to a decrease in plasma glucose to concentrations that trigger the secretion of autonomic counter-regulatory hormones, such as cortisol, adrenaline, growth hormone, and glucagon” [173,174,179]. Therefore, the effectiveness of lifestyle interventions on mental health based on the intensity and type of lifestyle can differ. In the studies included in this meta-analysis, there were differences in the lifestyle methods used, which affected the results of each study. Compared to other mental health interventions, lifestyle-based interventions may not be effective alone in improving mental health problems. Moreover, the effects of lifestyle interventions may not be achieved quickly, and therefore, other treatments, such as psychological and medicinal, also need to be considered. The effectiveness of lifestyle interventions on mental health is known [190], but how the costs and other aspects of this type of intervention compared to other psychological and pharmaceutical treatments compares need comparative study in the future.

Another significant finding was the effect of lifestyle interventions on depression, anxiety, and stress in women, confirming improvements across all three mental health domains. This study also revealed that the effectiveness of lifestyle interventions varied according to the scales used for assessment, with some yielding more significant results than others. Furthermore, the outcomes differed according to the patient population. For instance, depression showed a greater improvement among patients with metabolic disorders, or depression and cancer, whereas anxiety improved the most among those with depression and heart disease. These findings advocate lifestyle interventions as a component of comprehensive mental health care and highlight the need for public education on the connection between lifestyle and mental well-being.

### Strengths and Limitations

This study comprehensively reviewed common mental disorders, such as depression, anxiety, and stress, simultaneously in a systematic review and meta-analysis. In previous meta-analyses, different populations were not investigated. However, this distinction was made in this meta-analysis. This is because each population suffers from different diseases that can alter the effects of lifestyle interventions. Investigating gender differences was the study’s focus, and it was able to report results based on women separately; however, owing to the lack of studies, this review could not be performed for men. In addition, this study examined depression, anxiety, and stress based on different scales, which are the most important strengths of this meta-analysis. There are some limitations. These studies have primarily examined the effect of lifestyle interventions on depression and anxiety symptoms but not on depression and anxiety disorders, except for a few cases. Therefore, the generalization of the results to depression, anxiety, and stress disorders is limited. Each clinical trial on lifestyle has used different protocols and, although they have several commonalities, this heterogeneity might also impact the results. Variability in intervention types, such as the nature and intensity of lifestyle modifications, may affect the comparability of results across studies. Furthermore, the use of diverse measurement scales for mental health outcomes, although necessary for comprehensive analysis, introduces potential inconsistencies. Future studies could benefit from standardizing intervention protocols and measurement tools to enhance the comparability and robustness of their findings. Future studies should investigate the long-term impact of lifestyle interventions on mental health outcomes with an emphasis on their influence across broader demographic groups. Furthermore, analyzing subgroups, such as persons with diverse baseline mental health severities or differing socio-economic statuses, could provide more profound insights into the effectiveness and scalability of lifestyle interventions.

## 5. Conclusions

The findings showed the extent of the effectiveness of lifestyle-based interventions in improving mental health conditions, involving depression, anxiety, and stress. In addition, compared to other psychological and drug treatments, this type of intervention can be less expensive, healthier, and can be performed by more people. Therefore, considering and emphasizing these types of interventions can be highly beneficial and may have a long-term impact.

## Figures and Tables

**Table 2:** Lifestyle intervention on depression based on diseases.

Number of Studies	Disease	Hedges’s g	Lower Limit	Upper Limit	Z Value	*p*	*I* ^2^
**7**	Cancer	−0.34	−0.59	−0.08	−2.54	**0.011**	56.23%
10	Depression	−0.44	−0.62	−0.26	−4.82	**0.000**	40.46%
18	Diabetes/at risk of diabetes	−0.15	−0.27	−0.03	−2.43	**0.015**	56.51%
8	Heart-related disease	−0.19	−0.34	−0.04	−2.44	**0.015**	39.52%
6	Other mental disorders	−0.01	−0.17	0.15	−0.11	0.914	0%
2	Metabolic syndrome	−0.74	−1.27	−0.21	−2.76	**0.006**	69.66%
3	obstructive pulmonary disease	−0.14	−0.33	0.05	−1.44	0.151	0%
4	Older adults	−0.09	−0.23	0.05	−1.27	0.204	0%
4	Overweight/obesity	0.03	−0.19	0.24	0.25	0.802	53.18

**Table 3:** Lifestyle intervention on depression based on depression scales.

Number of Studies	Scale	Hedges’s g	Lower Limit	Upper Limit	Z Value	*p*	*I* ^2^
15	Beck Depression Inventory	−0.26	−0.45	−0.07	−2.62	**0.009**	73.07%
12	Center for Epidemiologic Studies Depression Scale	−0.23	−0.32	−0.14	−4.97	**0.000**	12.53%
14	Depression anxiety and stress scale	−0.15	−0.31	0.02	−1.76	0.078	49.69
4	Edinburgh Postnatal Depression Scale	−0.23	−0.58	0.13	−1.23	0.217	89.06%
3	Geriatric Depression Scale	−0.31	−0.71	0.10	−1.49	0.136	60.71%
19	Hospital anxiety and depression scale	−0.25	−0.35	−0.14	−4.62	**0.000**	30.95%
16	Patient Health Questionnaire	−0.16	−0.28	−0.05	−2.76	**0.006**	49.46%

## Data Availability

Data sharing is not applicable. No new data were created or analyzed in this study.

## Appendix A

**Table A1:** Keywords used for PubMed, Web of Science, Scopus, and the Cochrane Library and Scopus until August 2023.

Search	Query
PubMed	34,025
#1	Lifestyle intervention [Text Word] OR Lifestyle modification [Text Word] OR Lifestyle training [Text Word] OR Life Style [Mesh] OR Life Style [Text Word] OR Healthy Lifestyle [Mesh] OR Healthy Lifestyle [Text Word] OR Lifestyle change [Text Word] OR lifestyle behaviors [Text Word] OR Healthy Lifestyle Behaviors [Text Word]
#2	Agoraphobia [Mesh] OR Agoraphobia [Text Word] OR Neurotic Disorders [Mesh] OR Neurotic Disorders [Text Word] OR Obsessive-Compulsive Disorder [Mesh] OR Obsessive-Compulsive Disorder [Text Word] OR Hoarding Disorder [Mesh] OR Hoarding Disorder [Text Word] OR Phobic Disorders [Mesh] OR Phobic Disorders [Text Word] OR Social Phobia [Mesh] OR Social Phobia [Text Word] OR generalized anxiety disorder [Mesh] OR generalized anxiety disorder [Text Word] OR post-traumatic stress disorder [Mesh] OR post-traumatic stress disorder [Text Word] OR phobia [Mesh] OR phobia [Text Word] OR specific phobia [Mesh] OR specific phobia [Text Word] OR Panic Disorder [Mesh] OR Panic Disorder [Text Word] OR Obsessive-Compulsive [Mesh] OR Obsessive-Compulsive [Text Word] OR Neurosis [Mesh] OR Neurosis [Text Word] OR Obsessive-Compulsive Neurosis [Mesh] OR Obsessive-Compulsive Neurosis [Text Word] OR GAD [Mesh] OR GAD [Text Word] OR PTSD [Mesh] OR PTSD [Text Word] OR fear [Mesh] OR fear [Text Word] OR Panic [Mesh] OR panic [Text Word] OR anxiety [Mesh] OR anxiety [Text Word] OR Post-Traumatic [Mesh] OR Post Traumatic [Text Word] OR mental disorders [Mesh] OR mental disorders [Text Word] OR Stress [Mesh] OR Stress [Text Word] OR psychiatric disorders [Mesh] OR psychiatric disorders [Text Word] OR Mental illness [Mesh] OR Mental illness [Text Word] OR Depression [Mesh] OR Depression [Text Word] OR Depressive Symptom [Text Word] OR Depressive Disorders [Mesh] OR Depressive Disorders [Text Word] OR Depressive Syndrome [Text Word] OR Depressive Disorder, Major [Mesh] OR Depressive Disorder, Major [Text Word] OR Mood Disorders [Mesh] OR Mood Disorders [Text Word] OR Affective Disorders [Text Word] OR Common mental disorders [Text Word] OR Stress Disorders [Mesh] OR Stress Disorders [Text Word] OR Acute Stress Disorder [Text Word] OR Stress [Text Word] OR Stress, Physiological [Mesh] OR Stress, Physiological [Text Word] OR tension [Text Word]
Final	#1 AND #2
Scopus	24,470
#1	“Lifestyle intervention” OR “Lifestyle modification” OR “Lifestyle training” OR “Life Style” OR “Healthy Lifestyle” OR “Lifestyle change” OR “lifestyle behaviors” OR “Healthy Lifestyle Behaviors”
#2	“Agoraphobia” OR “Anxiety Separation” OR “Neurotic Disorders” OR “Obsessive-Compulsive Disorder” OR “Hoarding Disorder” OR “Phobic Disorders” OR “Social Phobia” OR “generalized anxiety disorder” OR “post-traumatic stress disorder” OR “phobia” OR “specific phobia” OR “Panic Disorder” OR “Obsessive-Compulsive” OR “Neurosis” OR “Obsessive-Compulsive Neurosis” OR “GAD” OR “PTSD” OR “fear” OR “panic” OR “anxiety” OR “Post-Traumatic” OR” mental disorders” OR “Stress” OR “psychiatric disorders” OR “Mental illness” OR “Depression” OR “Depressive Symptom” OR “Depressive Disorders” OR “Depressive Syndrome” OR “Depressive Disorder, Major” OR “Mood Disorders” OR “Affective Disorders” OR “ Common mental disorders” OR “Stress Disorders” OR “Acute Stress Disorder” OR “Stress” OR “Stress, Physiological” OR “Stress, Physiological” OR “Tension”
Final	#1 AND #2
Web of Science	26,395
#1	TS = (Lifestyle intervention OR Lifestyle modification OR Lifestyle training OR Life Style OR Healthy Lifestyle OR Lifestyle change OR lifestyle behaviors OR Healthy Lifestyle Behaviors)
#2	TS = (Agoraphobia OR Anxiety Separation OR Neurotic Disorders OR Obsessive-Compulsive Disorder OR Hoarding Disorder OR Phobic Disorders OR Social Phobia OR generalized anxiety disorder OR post-traumatic stress disorder OR phobia OR specific phobia OR Panic Disorder OR Obsessive-Compulsive OR Neurosis OR Obsessive-Compulsive Neurosis OR GAD OR PTSD OR fear OR panic OR anxiety OR Post-Traumatic OR mental disorders OR Stress OR psychiatric disorders OR Mental illness OR Depression OR Depressive Symptom OR Depressive Disorders OR Depressive Syndrome OR Depressive Disorder, Major OR Mood Disorders OR Affective Disorders OR Common mental disorders OR Stress Disorders OR Acute Stress Disorder OR Stress OR Stress, Physiological OR Stress, Physiological OR Tension)
Final	#1 AND #2
the Cochrane Library	5673
#1	Lifestyle intervention OR Lifestyle modification OR Lifestyle training OR Life Style OR Healthy Lifestyle OR Lifestyle change OR lifestyle behaviors OR Healthy Lifestyle Behaviors
#2	Agoraphobia OR Anxiety Separation OR Neurotic Disorders OR Obsessive-Compulsive Disorder OR Hoarding Disorder OR Phobic Disorders OR Social Phobia OR generalized anxiety disorder OR post-traumatic stress disorder OR phobia OR specific phobia OR Panic Disorder OR Obsessive-Compulsive OR Neurosis OR Obsessive-Compulsive Neurosis OR GAD OR PTSD OR fear OR panic OR anxiety OR Post-Traumatic OR mental disorders OR Stress OR psychiatric disorders OR Mental illness OR Depression OR Depressive Symptom OR Depressive Disorders OR Depressive Syndrome OR Depressive Disorder, Major OR Mood Disorders OR Affective Disorders OR Common mental disorders OR Stress Disorders OR Acute Stress Disorder OR Stress OR Stress, Physiological OR Stress, Physiological OR Tension
Final	#1 AND #2
